# Correction: Single-cell glycolytic activity regulates membrane tension and HIV-1 fusion

**DOI:** 10.1371/journal.ppat.1009584

**Published:** 2021-05-10

**Authors:** Charles A. Coomer, Irene Carlon-Andres, Maro Iliopoulou, Michael L. Dustin, Ewoud B. Compeer, Alex A. Compton, Sergi Padilla-Parra

After this article [1] was published, concerns were raised about the following results:

- In Fig 3A, the histograms below the beta-lactamase assay images appear similar.
- In Fig 4C, the upper vehicle panel and 100 μg/mL Chol panel appear to show the same image.

The authors noted that these duplications resulted from errors made when preparing the final figures. In reviewing the lab records, the authors identified additional data labelling errors in Fig 4C: the lower panels were mislabeled as to the experimental treatments represented in each. These issues are addressed in corrected figures provided with this notice.

In the original Fig 4C, the same Vehicle control (no cholesterol or 2-DG treatment) was meant to be reported in both the upper and lower rows as representing the same experimental condition. This duplication was removed from the corrected figure.

The original raw data were re-quantified to generate the updated histograms reported in the new Fig 4C; different analysis thresholds were applied in the reanalysis compared to the originally published analyses. Underlying data for the updated Fig 4C are in S5 File.

The underlying data for the Fig 3 and 4 experiments are in S1–S7 Files with this notice and at the Image Data Resource under accession number idr0103 (https://doi.org/10.17867/10000154).

Finally, there were errors in p value designations within the published figure legends. In Fig 1A statistical significance for two samples is indicated by *. The figure legend does not include this symbol but specifies, “*** p<0.001”. The figure legend should instead say “*p<0.05”. Also, in legends for Figs 3, 4, 6, S4, S6 and S7, * is listed as indicating p<0.5, this should instead say *p<0.05 in each instance. This is corrected in the updated Fig 3 and 4 legends with this notice, the same correction applies to the legends for Figs 6, S4, S6 and S7.

**Fig 3:**
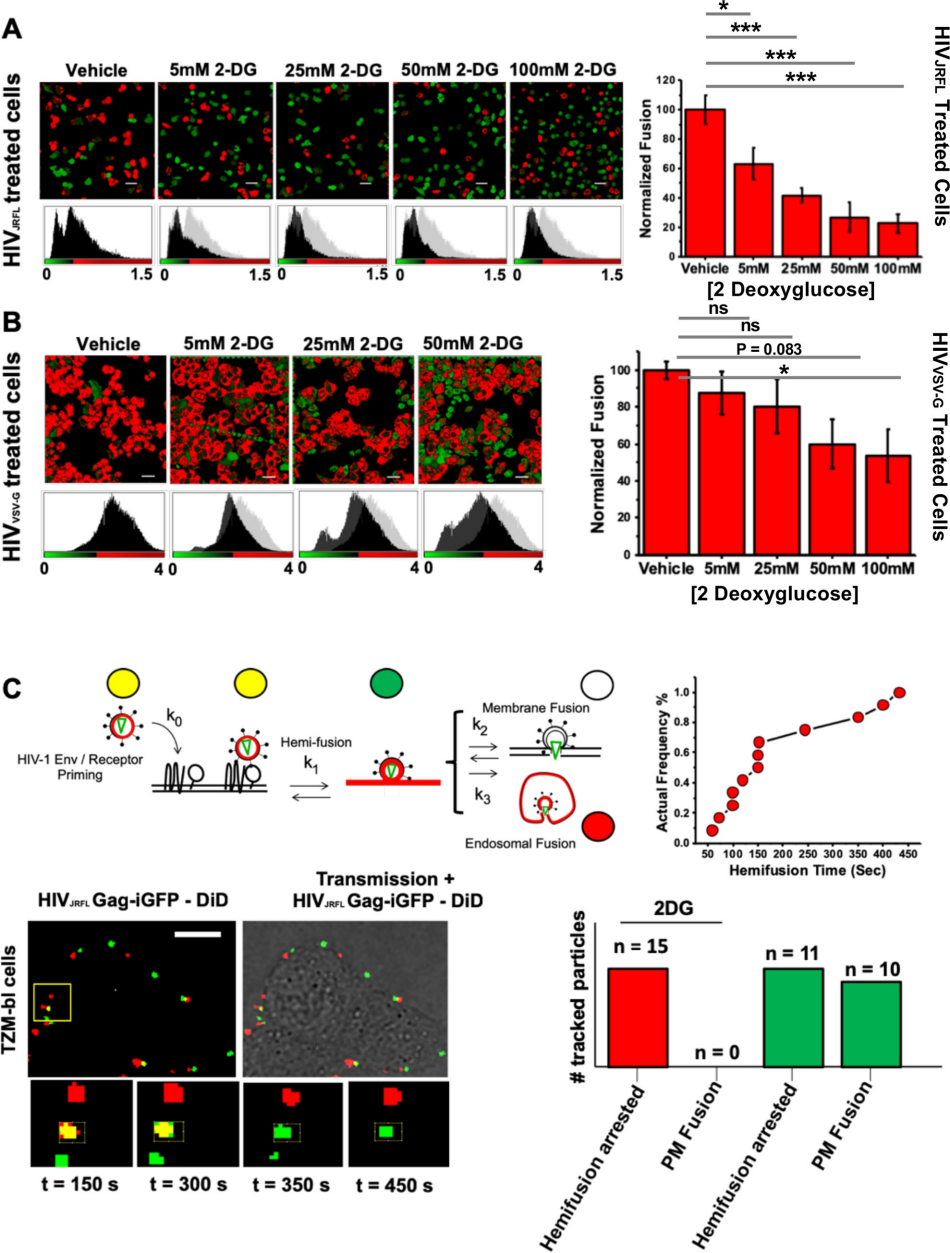
Addition of 2DG arrests HIV-1 fusion at the hemifusion stage. A.) (Left) Representative images of CCF2-loaded cells recorded 90 minutes after HIV-1_JR-FL_ infection in vehicle and incrementally increasing 2-DG treatment conditions; scale bar 50μm. (Right) Corresponding bar graph compiling data extracted from the β-lactamase assay and normalised to vehicle-treated control illustrating that increasing concentrations of 2-DG led to reductions in viral fusion for HIV-1_JR-FL._ in TZM-bl cells (mean of three independent experiments). *p<0.05, **p<0.01 *** p<0.001 as determined by one-way ANOVA. B.) (Left) Representative images of CCF2-loaded cells recorded 90 minutes after HIV-1_VSV-G_ infection in vehicle and incrementally increasing 2-DG treatment conditions; scale bar 50μm. (Right) Corresponding bar graph compiling data extracted from the β-lactamase assay and normalised to vehicle-treated control illustrating that increasing concentrations of 2-DG led to partial reduction in viral fusion for HIV-1_VSV-G_ in TZM-bl cells (mean of three independent experiments). *p<0.05, **p<0.01 *** p<0.001 as determined by one-way ANOVA. In panels A and B, the control histogram data are overlaid as gray curves in the experimental histogram panels to enable easy comparison of the results. C.) (Top row, left) Cartoon diagram illustrating the concept of single-particle tracking with double-labelled virions with DiD and eGFP-gag. Briefly, double-labelled virions entering via endocytosis will have their eGFP-gag signal infinitely diluted during endosomal fusion whilst DiD signal is retained in the endosome, which is mobile. Virions entering via plasma membrane fusion will have their DiD signal infinitely diluted in the plasma membrane whereas the eGFP-gag signal is retained and mobile. Hemifusion is denoted when DiD signal infinitely diluted in the plasma membrane whereas the eGFP-gag signal is retained and immobile. (Top row, right) Kinetics of the individual hemifusion events plotted as cumulative distributions as a function of time. (Bottom row, left) Representative panel of images illustrating doubled-labelled HIV-1_JRFL_ particles losing DiD signal (red) and maintaining immobile eGFP signal (green) when attempting fusion in 2-DG treated cells, suggesting arrest at hemifusion (n = 15, acquired during three independent experiments). (Bottom row, right) Bar chart representing the total number of HIV_JRFL_ double-labelled particles tracked for TZM-bl cells treated with 2DG (red bars) and without treatment (green bars). Only in cells without 2-DG treatment plasma membrane fusion was observed. Total number events tracked in control conditions: 217. Total number of events tracked in 2-DG-treated conditions: 236.

**Fig 4:**
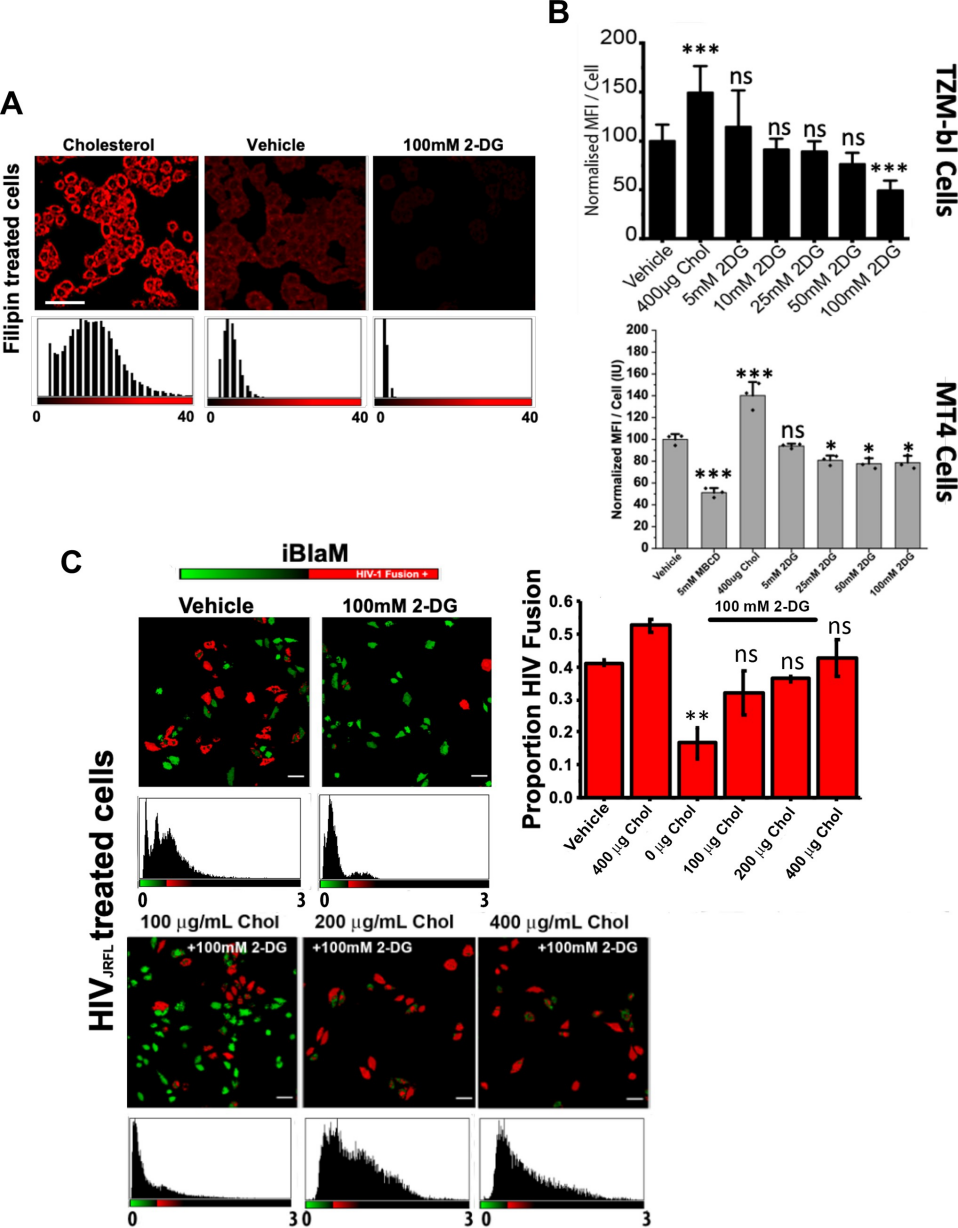
Addition of 2-DG sequesters cholesterol from the cell membrane. A.) Representative images depicting filipin mean fluorescence intensity per cell indicating two-hour incubation of increasing concentrations of 2-DG decreases surface cholesterol in TZM-bl cells; scale bar 50 μm. B.) Bar charts depicting mean fluorescence intensity per cell of three independent experiments normalised to vehicle in TZM-bl cells (top) and MT4 cells (bottom) in cells treated with 400μg/mL cholesterol, 5mM MBCD and increasing concentrations of 2-DG. C.) Representative images (left) and bar chart (right) of three independent experiments depicting percent of fusion positive cells, as determined by the BlaM assay, relative to vehicle control illustrating increasing concentrations of cholesterol rescues fusion in 2-DG treated TZM-bl cells; scale bar 50 μm. In the histogram for Fig 4C, ‘μg/mL’is the correct unit of cholesterol (Chol) concentration, but this is abbreviated to ‘μg’ in the x axis data labels. The x axes in the histograms represent the relative values in arbitrary intensity units of the ratio between the blue and the green channels (B/G). *p<0.05, **p<0.01 *** p<0.001 as determined by one-way ANOVA.

The *PLOS Pathogens* Editors confirmed that the updated figures support the results statements reported in the published article.

## Supporting information

S1 File Underlying data to support quantitative results reported in Fig 3A, including all statistical analyses for cells treated with 2DG. The bar graph in the figure is graph 11 in this file.
(OPJ)

S2 File Information about how raw image data were processed to generate the results shown in Fig 3A. The same method was used to process the data reported in Fig 4C, except that different limits were applied in inverting colors (0–0.9 in Fig 3A, 0–0.8 in Fig 4C), and a different dynamic range was used in obtaining the histograms (0–1.5 in Fig 3A versus 0–3 in Fig 4C).
(PDF)

S3 File Raw image data and histograms underlying Fig 3A. (ZIP)

S4 File Raw data and graphs supporting the quantitative results reported in Fig 4C. Graph 5 in this file corresponds to the bar graph shown in the published figure.
(OPJ)

S5 File Raw image data and histograms underlying updated Fig 4C. The corresponding author noted that the histograms in the updated figure differ from those in the published article and were obtained from the same raw image data but applying different analysis thresholds.
(ZIP)

S6 File Underlying data for other panels of Fig 3. (ZIP)

S7 File Underlying data for Fig 4A and 4B. (ZIP)
