# Supplementary figures and images for: Single-cell glycolytic activity regulates membrane tension and HIV-1 fusion
Source: PLoS Pathog. 2020 Feb 21;16(2):e1008359. doi: 10.1371/journal.ppat.1008359 (PMC7055913; doi:10.1371/journal.ppat.1008359)

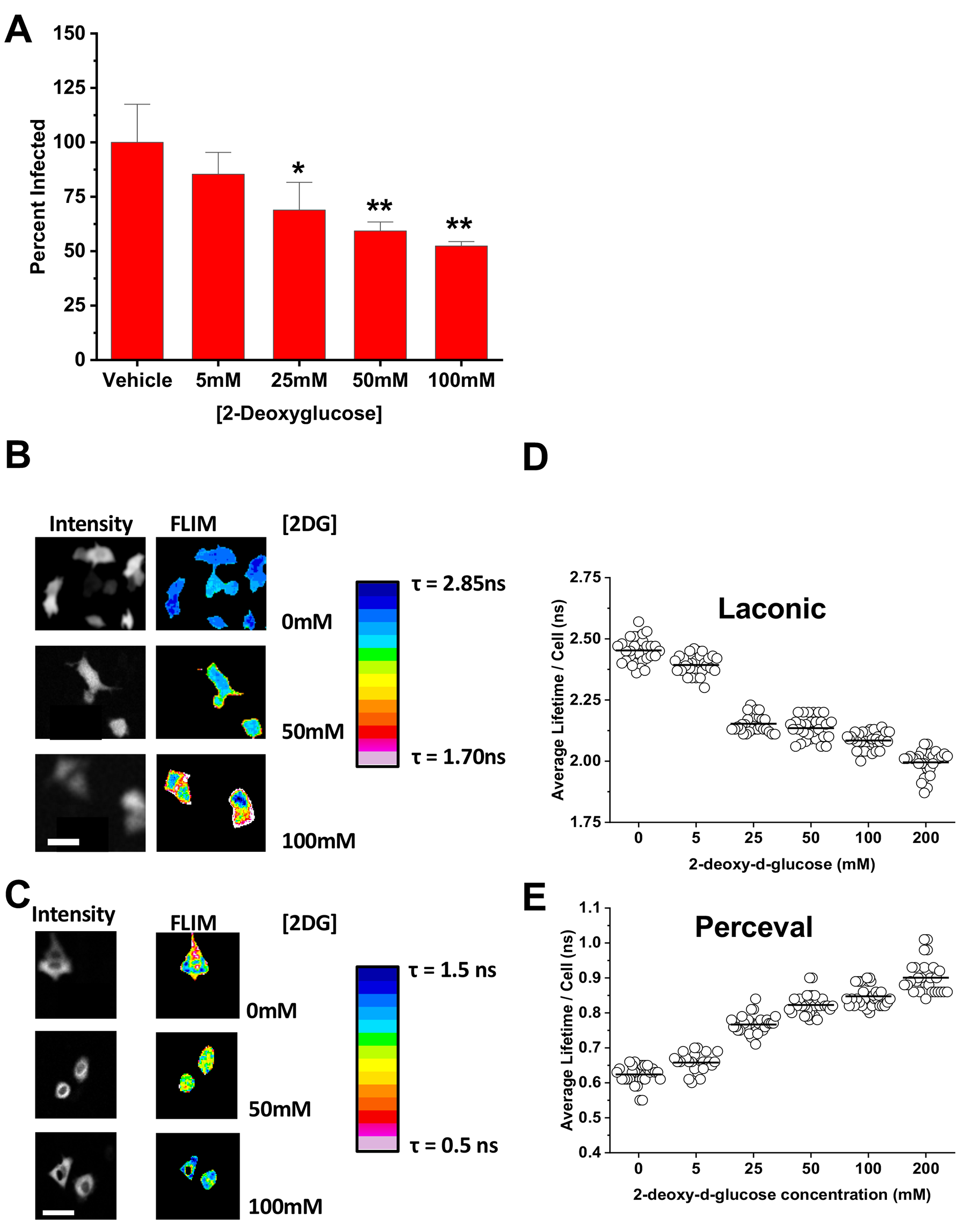

Supplement: S1 Fig — A.). Bar charts depicting % eGFP-expressing cells as a marker of infection illustrating that acute treatment with increasing concentrations of 2-DG led to reductions in HIV-1NL4.3 infection in human MT4 T cells. B.) Representative intensity (left) and fluorescent lifetime imaging (right) of single cells transiently expressing intracellular lactate biosensor Laconic in single TZM-bl cells with increasing concentrations of glycolytic inhibitor 2-DG; scale bar, 50μm. C.) Representative intensity (left) and fluorescent lifetime imaging (right) of single cells transiently expressing intracellular ATP:ADP ratio biosensor Perceval in single TZM-bl cells with increasing concentrations of glycolytic inhibitor 2-DG; scale bar, 50μm. D.) Dot plots representing lifetimes of intracellular lactate biosensor Laconic extracted from live, single cells as regions of interest post-treatment for 2-hours with increasing concentrations of 2-DG in TZM-bl cells. E.) Dot plots representing lifetimes of intracellular ATP:ADP ratio biosensor Perceval extracted from live, single cells as regions of interest post-treatment for 2 hours with increasing concentrations of 2-DG in TZM-bl cells. (TIF) [file ppat.1008359.s001.tif]

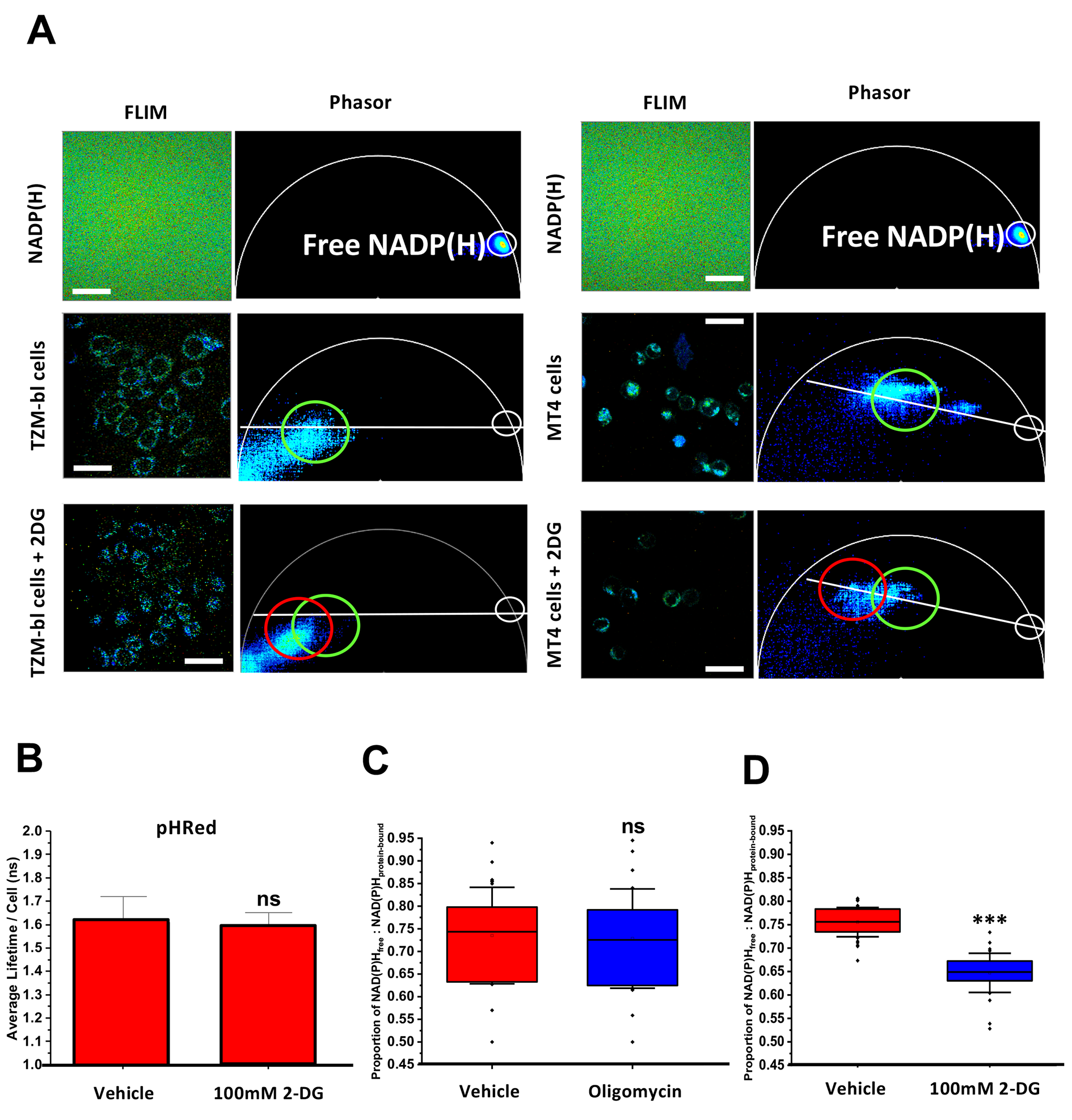

Supplement: S2 Fig — A.) Representative images (left) and phasor plots (right) representative of FLIM distributions of NAD(P)H alone (top row), vehicle-treated conditions in TZM-bl (left) and MT4 cells (right) (middle row) and acute treatment with 2-DG (bottom row); scale bar 5 μm. Phasor FLIM plots illustrate each pixel converted via Fourier Transform to the phase domain. The phasor plots illustrate longer lifetimes (i.e. enzyme-bound NAD(P)H, lower glycolytic flux) to the left and shorter lifetimes (free NAD(P)H, higher glycolytic flux) to the right. B.) Bar charts representing the lifetime extracted from single TZM-bl cells expressing intracellular pH biosensor pHRed indicating a lack of change in fluorescence lifetime during acute 2-DG treatment. C.) Box plot representing the ratio of NAD(P)Hfree vs. NAD(P)Hprotein-bound in MT4 cells treated with oligomycin. D.) Box plot representing the ratio of NAD(P)Hfree vs. NAD(P)Hprotein-bound in MT4 cells treated with 2-DG. The presented two-photon FLIM data was acquired as described in material and methods. Box plots represent data acquired from at least 30 cells per condition acquired from three independent experiments.*** p<0.001 as determined by one-way student’s T-test. (TIF) [file ppat.1008359.s002.tif]

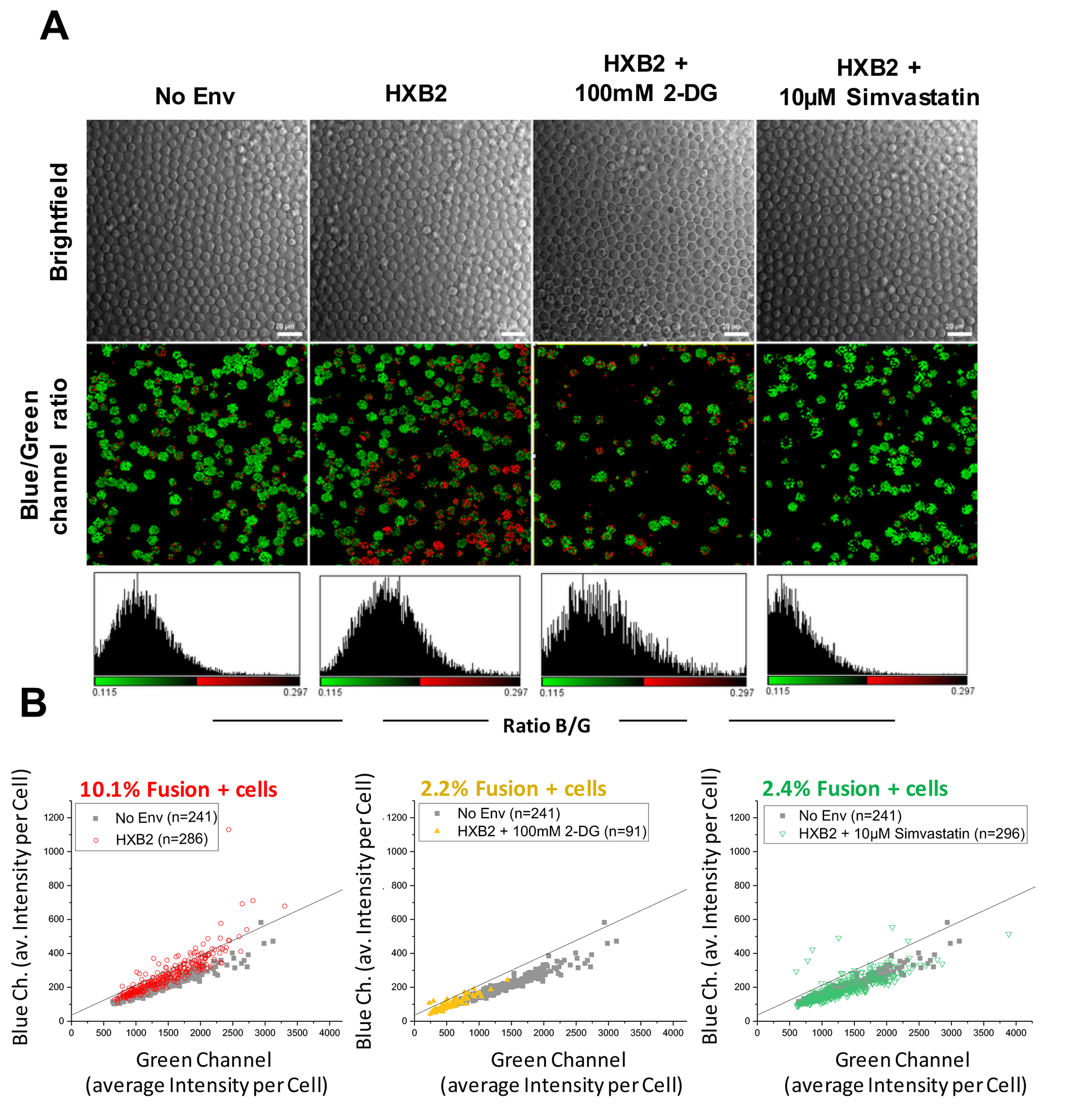

Supplement: S3 Fig — A) Primary cells were exposed to either naked (i.e. No Env) HIV-1 or HIV-1HXB2 virions and treated with vehicle, 100 mM 2-DG or 10μM Simvastatin. Brightfield images (first row) show that in all cases the integrity of the cells was maintained. The BlaM assay for HIV fusion (Blue/Green channel ratio images, second row) shows that the number of positive fusion cells (red) is higher for cells only exposed to HIVHXB2. Cells exposed to both HIVHXB2 and 100 mM 2-DG or 10uM Simvastatin were less susceptible to HIVHXB2 fusion as limited fusion positive cells (red cells) were detected. The pixel-by-pixel histograms for each condition are also shown for each condition in the lowest row. B) When quantifying the overall populations of cells (i.e. at least100 cells per condition) and taking as a negative control No Env HIVHXB2 (gray dots, and straight gray line) as a threshold for fusion, one could see that in the green channel versus blue channel plots (based in average intensities recorded from single cells) 10.1% of primary T cells were fusion positive when exposed to HIVHXB2 (red dots above the grey No Env threshold line in the left panel). For cells treated with 100 mM 2-DG, only 2.2% turned out to be fusion positive (red dots above the grey line in the middle panel). In turn, for cells treated with 10μM Simvastatin, only 2.4% were fusion positive (green dots above the grey No Env threshold line, right panel). (TIF) [file ppat.1008359.s003.tif]

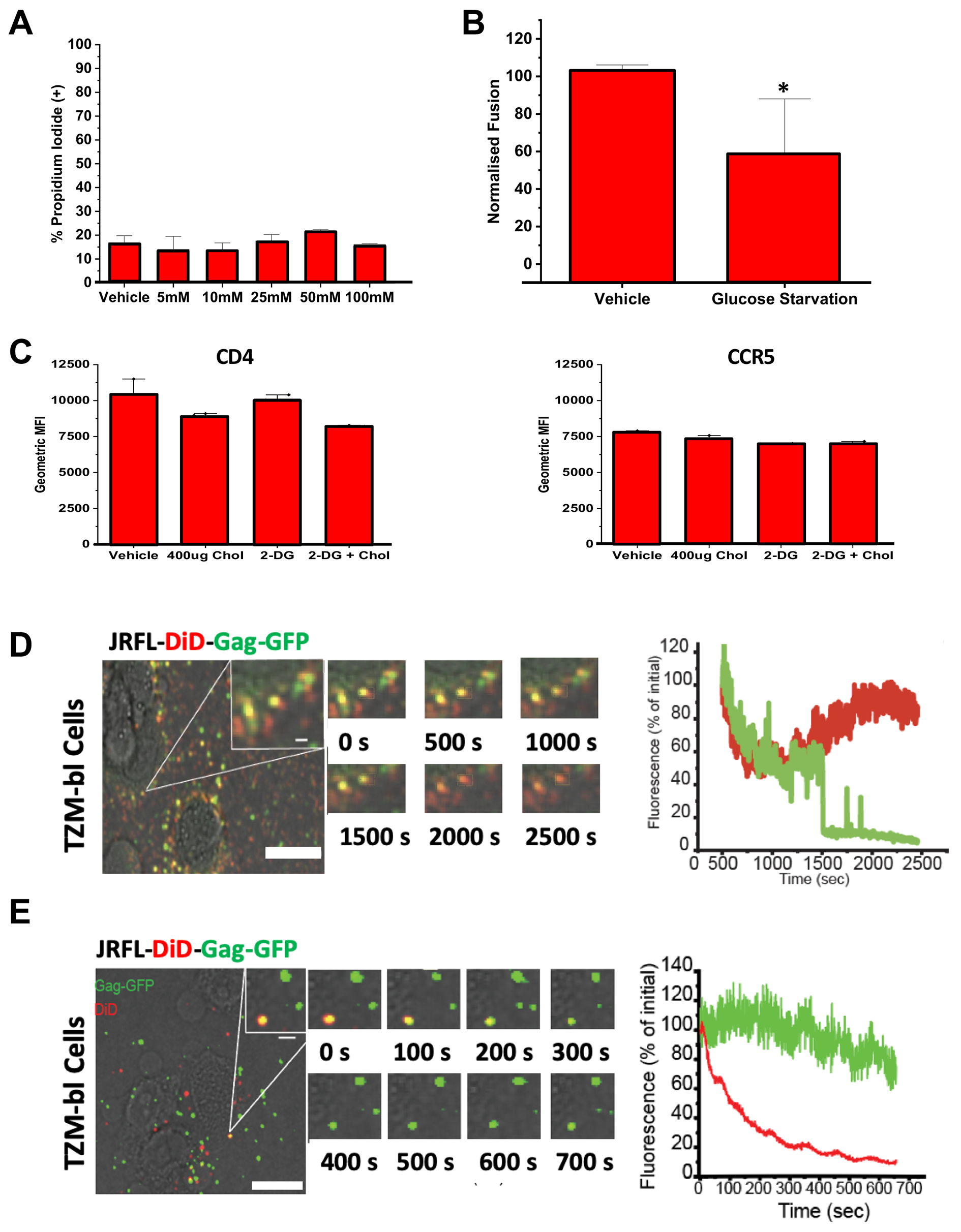

Supplement: S4 Fig — A.) Bar charts depicting the percentage of dead TZM-bl cells detected by propidium iodide (PI) staining in single cells treated with increasing concentrations of 2-DG for two hours. B.) Bar charts representing normalized HIV-1JR-FL fusion relative to vehicle in single TZM-bl cells as determined by the β-lactamase assay in cells treated with glucose-free medium for two hours before virus addition. C.) Bar charts illustrating that relative CD4 and CCR5 expression levels do not drastically change during listed treatment conditions. Bar charts shown in the panel are representative of a mean of three independent experiments. D.) Representative fluorescence series of images (left) and single particle tracking traces (right) of Gag-eGFP (green) and DiD (red) dual-label HIV-1JR-FL pseudovirions in TZM-bl cells (scale bar) illustrating that in control conditions (i.e. no 2-DG) that HIV-1JR-FL entry in TZM-bl cells proceeds with a precipitous loss of Gag-eGFP and maintenance of DiD signal, indicative of endocytosis as previously described (n = 15, acquired during three independent experiments). E.) Representative fluorescence series of images (left) and single particle tracking traces (right) of Gag-eGFP (green) and DiD (red) dual-label HIV-1JR-FL pseudovirions in TZM-bl cells (scale bar) illustrating that in 2-DG-treated conditions (i.e. 100mM 2-DG) that HIV-1JR-FL entry in TZM-bl cells may also proceed with a slow decay of DiD signal and maintenance of eGFP-Gag signal, indicative of hemifusion (n = 15, acquired during three independent experiments). *p<0.5, **p<0.01 *** p<0.001 as determined by either one-way ANOVA or one-way student’s T-test. (TIF) [file ppat.1008359.s004.tif]

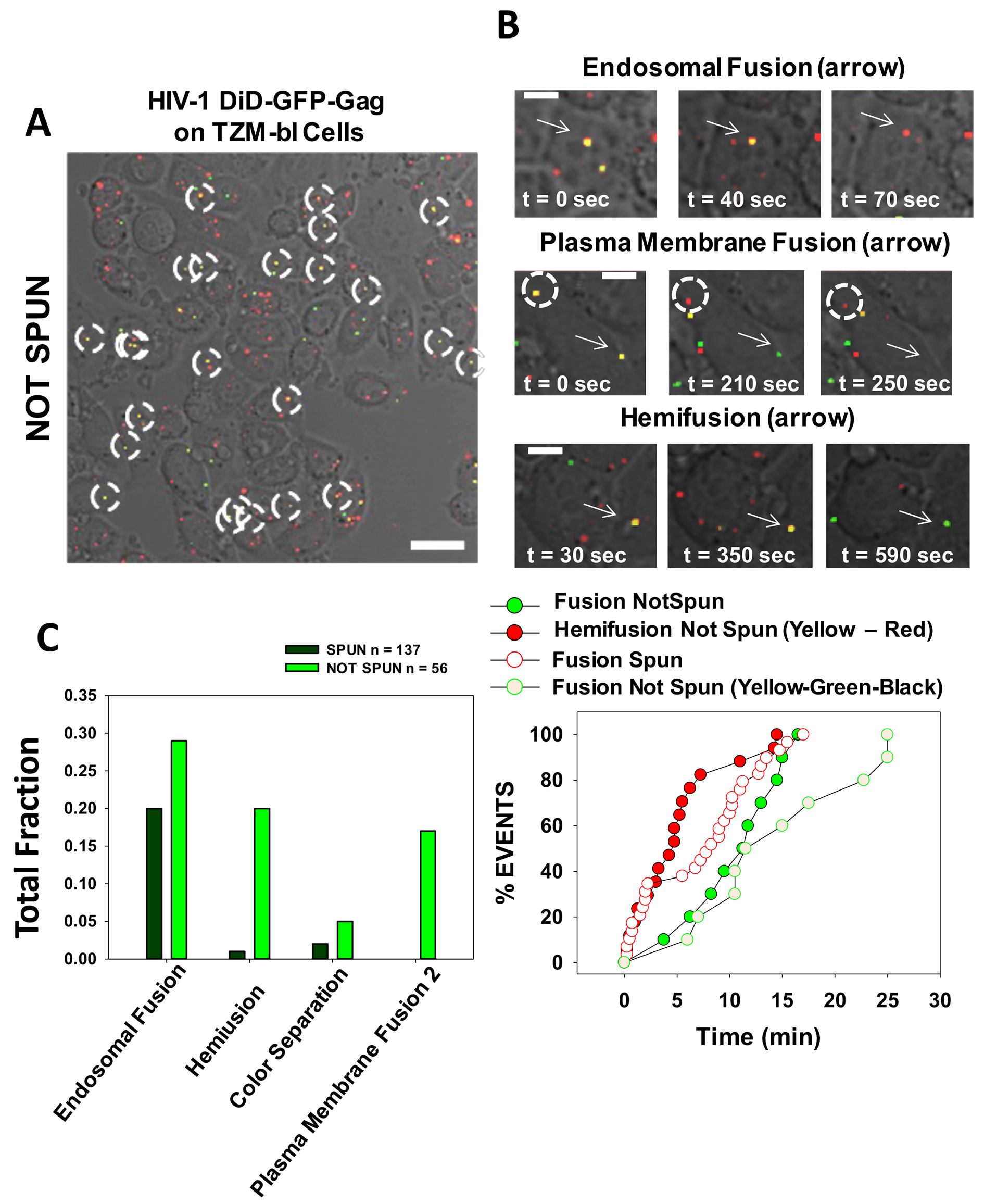

Supplement: S5 Fig — A.) HIVJR-FL pseudoviruses labelled with DiD and Gag-GFP exposed on TZM-bl at 4°C during 30 min as described in material and methods were placed under the microscope at 37°C to start the entry process. White dotted circles indicate the HIV-1 particles co-labelled with both colours (red for DiD and green for Gag-GFP). A subset of these particles are only labelled with either Gag-GFP (green dots) or DiD (red dots). These particles were ignored. Scale Bar = 10 μm B.) Three regions of interest showing: i) endosomal fusion (first row, where a yellow particle turns red signifying DiD redistribution within the endosomal compartment and Gag-GFP release); ii) plasma membrane (PM) fusion (second row, where a double labelled particle designated with an arrow first changes from yellow to green indicating hemifusion (i.e. DiD dilution on the PM) and then Gag-GFP disappearance. A white dotted particle undergoing endosomal fusion is also shown indicating that the focus was kept stable during the acquisition and also that both phenomena can occur in non-spinoculated cells simultaneously. The third row shows a particle undergoing hemifusion (unable to progress toward full fusion as the GFP-Gag remains through time). Scale bar = 1 μm C.) Left panel: The statistics for spun (dark green) versus non-spun (light green) cells are shown. The proportion of events (0 to 1) for endosomal fusion, hemifusion, colour separation (capsid release) and plasma membrane fusion are shown for a total of n = 137 and n = 56 double labelled particles for spun and non-spun TZM-bl cells respectively. Also, a small fraction of events for both spun and not-spun conditions showed colour separation; colour separation is defined as double-labelled (yellow viruses) internalized within endosomes (labeled with DiD and Gag-GFP) undergoing fusion. Right at the moment of fusion DiD redistributes around the endosomal membrane and partially uncleaved Gag-GFP is released into the cytosol.The right panel depicts the cumula [file ppat.1008359.s005.tif]

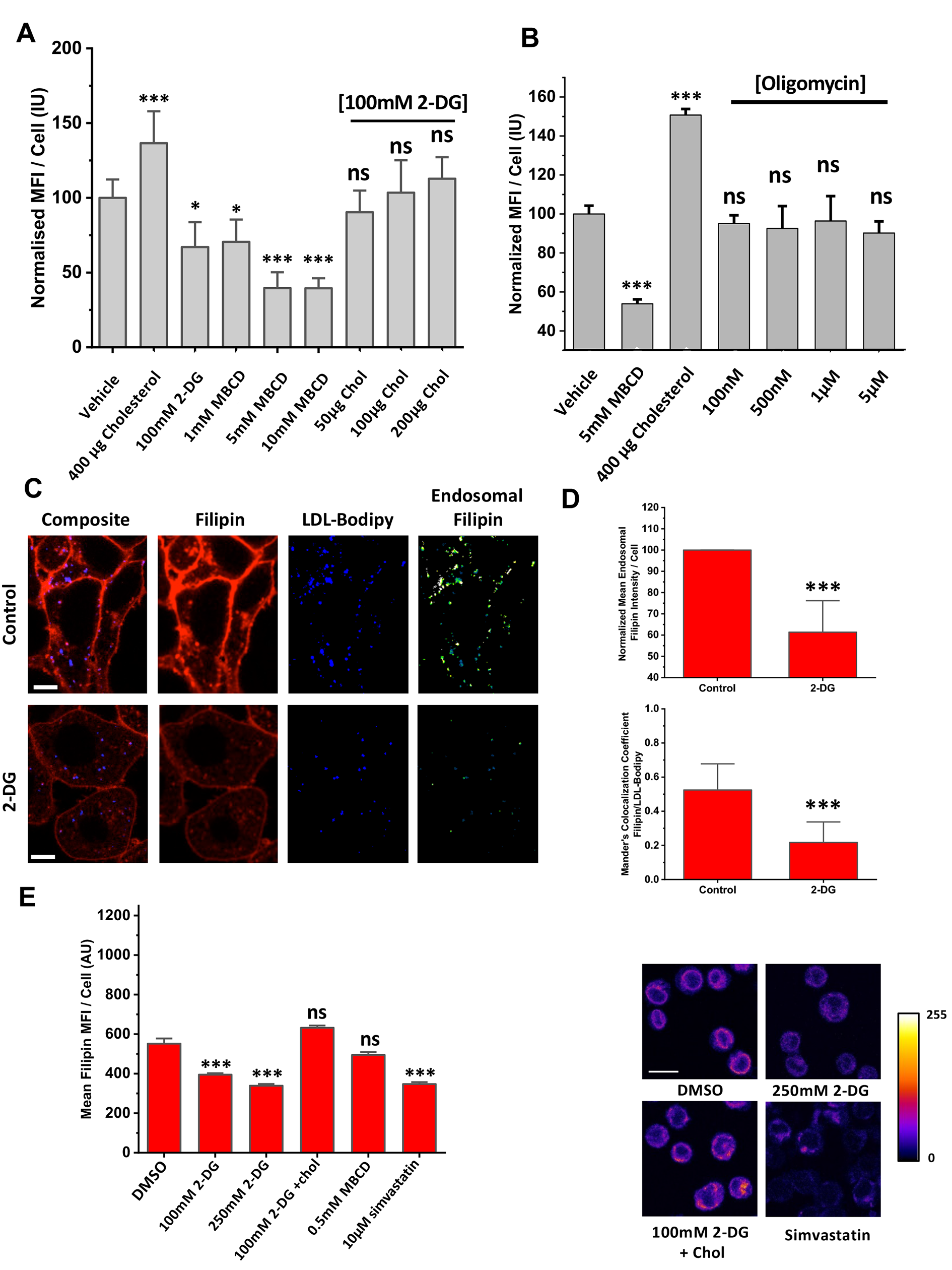

Supplement: S6 Fig — A.) Bar charts depicting normalized filipin mean fluorescence intensity per cell in various treatment conditions listed to illustrate that 2-DG treated cells have plasma membrane cholesterol content similar to 1mM MBCD-treated cells and that supplementation with increasing concentrations of water-soluble cholesterol in 2-DG treated conditions rescues filipin staining signal. B.) Bar charts depicting filipin mean fluorescence intensity per MT4 T cell normalised to vehicle, indicating two-hour incubation of increasing concentrations of oligomycin do not alter surface plasma membrane cholesterol. C.) TZM-bl cells were grown in vehicle (top) or 2-DG-treated (bottom) conditions for two hours after being loaded with LDL-Bodipy staining to label the endosomal compartment. Filipin staining was used to label cholesterol at both the cell surface (red) and LDL-Bodipy (blue) identified regions (green illustrates the LDL-Bodipy associated cholesterol identified by LDL-Bodipy and filipin co-labelling); scale bar 10μm. D.) Bar charts illustrating normalized filipin mean fluorescence intensity in LDL-Bodipy labelled compartments in vehicle and 2-DG-treated conditions (top) and colocalization coefficients of LDL-Bodipy with filipin quantified by z-stacks (bottom). E.) (Left) Bar charts depicting raw filipin mean fluorescence intensity per primary CD4+ T cell in each condition listed. (Right) Representative images of primary CD4+ T cells stained with filipin after treatment conditions listed. Mean values were calculated from at least 50 cells per condition from three independent experiments. *p<0.5, **p<0.01 *** p<0.001 as determined by either one-way ANOVA or one-way student’s T-test. (TIF) [file ppat.1008359.s006.tif]

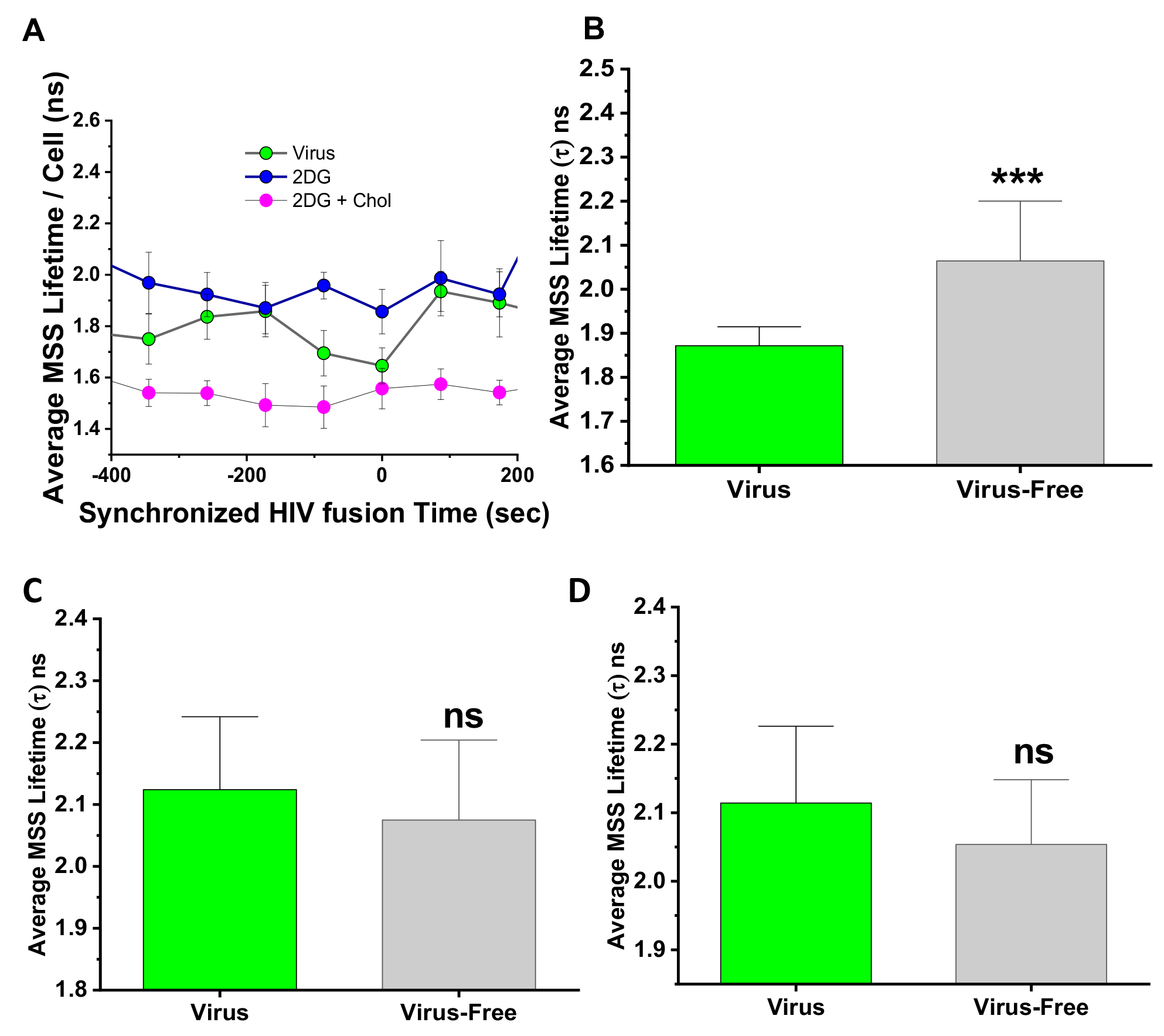

Supplement: S7 Fig — A.) Compiled MSS lifetimes extracted from whole TZM-bl cells compiled from acquisitions of multiple viral fusion events (green) compared to 2-DG treated cells where viruses were incapable of fusion (blue) or rescued events due to cholesterol treatment (purple). Each point represents a mean of at least 20 cells obtained during three separate experiments. B.) Bar charts comparing the MSS lifetimes of localised regions overlapping with mCherry-labelled HIV-1JR-FL during virion fusion (i.e. mCherry signal loss) with virus-free control areas in the same cell at the timepoint of fusion in TZM-bl cells. Each condition represents a mean of at least 20 cells obtained during three separate experiments *p<0.5, **p<0.01 *** p<0.001 as determined by either one-way student's T-test C.) Bar charts comparing the MSS lifetimes of localised regions overlapping with mCherry-labelled HIV-1JR-FL (i.e. mCherry signal) with virus-free control areas in the same cell at the timepoint before fusion in TZM-bl cells. Each condition represents a mean of at least 20 cells obtained during three separate experiments *p<0.5, **p<0.01 *** p<0.001 as determined by either one-way student's T-test. D.) Bar charts comparing the MSS lifetimes of localised regions overlapping with regions where mCherry-labelled HIV-1JR-FL fused with the host cell (i.e. mCherry signal lost) with virus-free control areas in the same cell at the timepoint after fusion in TZM-bl cells. Each condition represents a mean of at least 20 cells obtained during three separate experiments *p<0.5, **p<0.01 *** p<0.001 as determined by either one-way student's T-test. (TIF) [file ppat.1008359.s007.tif]
