# Supplementary material for: Single-cell glycolytic activity regulates membrane tension and HIV-1 fusion
Source: PLoS Pathog. 2020 Feb 21;16(2):e1008359. doi: 10.1371/journal.ppat.1008359 (PMC7055913; doi:10.1371/journal.ppat.1008359)
Supplement: Attachment — Submitted filename: Rebuttal Coomer et al-2019-FINAL.docx [file ppat.1008359.s008.docx]

Cholesterol in target membranes enhances diverse membrane fusion events, and in several cases, this has been shown to reflect a change in membrane tension. For example, both factors (cholesterol and membrane tension) have been shown to be important for HIV fusion. Investigators have also previously shown that the cell's metabolic state is a determinant of HIV infection. Here, the authors argue that these properties are linked, and attempt to characterize the linkage between ATP/lactate levels, plasma membrane cholesterol, membrane order and tension, and HIV fusion and infection. They do so employing FLIM (fluorescence lifetime imaging microscopy), FRET-based biosensors, and multi-color single virus particle tracking. The findings extend prior observations and the imaging experiments are generally state of the art, but considerable additional experimentation would be required to compellingly demonstrate that metabolic activity dictates HIV infectibility through cholesterol-dependent changes in membrane tension, explain how endosomal fusion is affected by changes in plasma membrane cholesterol levels, and demonstrate that these are biologically important phenomena.

**Significant issues**

1. -In figure 1, the authors state that "2-DG resulted in dose-dependent decreases in the relative amounts of lactate and ATP:ADP and a concomitant inhibition of HIV-1JR-FL fusion (Figure 3A and 3B)." 2-DG data for lactate and ATP:ADP appear to be presented in supplemental Figure 1A/1B. However, the data presented show a decreased "average lifetime/cell" of Perceval and an increased "average lifetime/cell" for Laconic (Why does it increase?). ATP:ADP ratios and intracellular lactate levels in 2-DG-treated cells do not appear to be presented in a manner equivalent to that of Figure 1D or 2B/2C. How do the values in 2DG-treated cells compare with control MT4 cells that are "Fusion+" and "Fusion-"?

*We appreciate the reviewer’s concern and appreciate the confusion that can stem from the lifetime data in supplemental Figure 1A/1B. The two reporters utilised in this study have been previously published on and characterized, so we would first like to refer the reviewer to these studies for a more thorough characterization of these reporters. However, in brief, these reporters (i.e. Perceval and Laconic) detect their metabolites in fundamentally different ways, such that their graphical trends are not necessarily comparable. Perceval’s (i.e. ATP/ADP ratio sensor) is a circularly permuted biosensor whose lifetime increases upon decreasing ATP concentrations and increasing ADP concentrations. This peculiar change is due to the lack of ATP in the biosensor’s binding pocket, changing the conformation of the sensor, and thus making FRET less likely to occur, increasing the donor lifetime. Therefore, when ATP concentrations are high, the lifetime is low (high FRET) and when ATP concentrations are low, (low FRET) the lifetime is high. Due to fundamental differences in the metabolic reporter Laconic’s structure, the situation is different here. Laconic directly binds intracellular lactate as described. However, upon doing so, structural changes in the biosensor increase the distance between the donor and acceptor fluorophores, therefore making FRET less likely to occur and therefore increasing the lifetime of the fluorophore. Consequently, high lactate concentrations increase the lifetime. We have added a brief explanation in lines 128-132 and specifically refer the reader to the literature in order to help guide him/her in interpreting lifetime data in the context of metabolic biosensors*

*Unbeknownst to the reviewers, we mislabelled the graphs (i.e. Perceval was labelled as “Laconic” and “Laconic” was labelled “Perceval” in supplemental figure 1A/1B. We have switched these to their correct positions and hope the above explanation aids the reviewer.*

*The reviewer has correctly pointed out that we have presented the Perceval and Laconic in supplemental figures 1A/1B in a slightly different manner when compared to Figure 1D and Figure 2B/C. These are intentionally different for specific reasons. Supplemental figure 1A/1B is utilized to specifically illustrate raw lifetime values (i.e. in nanoseconds) so the reader can appreciate that decreasing concentrations of ATP or lactate can actually cause physical changes to the sensor, thus altering their lifetimes, in the context of 2-DG treatment. As explained explicitly in the methods, which we have now emphasized in lines 136-137, we utilized these lifetime values and associated amplitudes from their decay curves to directly calculate relative concentrations of either metabolite. We hope this explanation clarifies this to the reviewer.*

*The reviewer has made an interesting final comment here asking how these values of ATP/ADP ratios and relative lactate concentrations change upon 2DG treatment in MT4 cells. Unfortunately, this question is not straightforward to answer in T cells given the difficulty of transfection and expression of these biosensors in primary cells and cell lines, which we have tried and can send this data upon request. To circumnavigate this problem, we utilized two photon microscopy to calculate NAD(P)/NAD(P)H (Blacker and Duchen, Free Radic Biol Med. 2016; Gomez et al. PLoS One, 2018; Schaefer et al. Cytometry. 2018) values to aid in this endeavour and we now include in the supplemental figure (i.e. see supplemental figure 1G). As provided in the references and the review article, NAD(P)H autofluorescence evaluation by two-photon FLIM is now a well-accepted methodology to evaluate the bioenergetic state of the cell (i.e. majority glycolysis or majority oxidative phosphorylation). Indeed—treatment of MT4 cells with 2DG acutely leads to a change in the MT4 NAD(P)H_free_ /NADPH_protein-bound_ lifetime ratio as, indicating a shift in the redox ratio, lower concentration of free NADH, indicating a decrease in glycolytic flux and metabolic activity. The associated text for this methodology appears in lines 133-136.*

2) More information is needed to justify the conclusion that fusion mediated by HIV Env is more sensitive to metabolic state than fusion mediated by VSV-G. Line 149 states (citing Fig. 3A) that 100 mM 2DG pre-treatment caused ~80% inhibition HIV fusion. However, in Supplemental Fig. 1A, 100 mM 2DG 2DG caused only ~50% inhibition of HIV infection. For VSV-G-HIV pseudoviruses, 100 mM 2DG caused ~50% inhibition of both fusion (Fig. 3B) and infection (Fig. 1A).

*We appreciate the reviewers for making this critical comment, and we recognize that this conclusion stating that HIV JRFL is more sensitive to the host metabolic state than VSV-G was unsuitably made given the present data. We have removed this conclusion from the body of the text and would like to note that this conclusion does actually not impact the main message of the paper (i.e. metabolic activity of the host cell dictates HIV infectibility through cholesterol-dependent changes in membrane tension). Our primary aim from this additional experiment (i.e. using VSV-G) was to have an alternative envelope to test and make sure our results were not exclusive to HIV envelopes. We will move this image to the supplemental figure accordingly and explain this in the text.*

*In regards to the issue with line 149, we recognize that the reviewer may be alarmed by this discrepancy, however, this is an explainable result. Initially, it should be noted that the data between supplemental Fig. 1A and Figure 3 are not, and should not be, compared for multiple reasons. First, supplemental Figure 1A was created to re-iterate claims made in prior studies (i.e. Valle-Casuso et al 2018) that metabolic activity is critical in determining HIV-1 infection in T cells, and that glycolytic arrest with 2-DG can reduce HIV-1 infection in T cells. To do this, we utilized MT4 cells which are susceptible to NL4.3 Env, an X4 tropic virus. Because our results suggested that acute arrest in glycolysis leads to an inhibition of NL4.3 infection, we switched our cell line of choice (i.e. TZM-bls) to utilise microscopy-based approaches to investigate this phenomenon, which are highlighted in the rest of the paper.*

*Consequently, results obtained in Supp. Figure 1A may not be comparable to Figure 3, which utilises TZM-bl cells and necessary R5-tropic JR-FL. We settled on this approach as we wanted to balance a primary isolate (Figure 3, JR-FL) to provide a more physiologically-relevant isolate in the study, whilst still being able to maintain the microscopy-based systems utilized in Figure 1 and 2 to extend their results, as well as utilize further microscopy approaches throughout the rest of the manuscript. As such, given that the cell lines and viruses utilized in the manuscript are different from supplemental figure 1A to Figure 3, it is not surprising that differences of ~20% are obtained. We have eliminated any notion of comparisons (i.e. lines 169-170 and 339-340 between these two figures and have explicitly emphasized the role of each cell line and virus used in the text when appropriate to ameliorate this controversy.*

*Additionally, the results obtained from supplemental Figure 1a and Figure 3 were acquired by different mechanisms (i.e. flow cytometry and microscopy BLaM, respectively). As such, these methods should not be compared given the sensitivity between both assays are different and subject to different biases (i.e. flow cytometry being a bulk assay whereas each individual cell can be scrutinized in microscopy BlaM). Additionally, the types of biases between both assays are different. For example, false-positives in bulk assays like flow cytometry (i.e. from auto-fluorescence or from eGFP-tagged virions sticking to the surface of cells) could also create a higher infection rate than true value. This could artificially raise the %-infected cells here, further adding to this discrepancy.*

a. For Fig. 3A and 3B, were viral inputs (e.g., p24 added/cell) of HIV-JFL and HIV-VSV-G normalized? The cells in Fig. 3B appear denser than in Fig. 3A. For further assurance, the authors should directly compare entry of pseudoviruses bearing HIV Env and pseudoviruses bearing VSV-G (with normalized inputs of pseudoviruses and the same set of cells). They should also provide the raw %entry values in the vehicle samples that are used to normalize the data. (Were there similar levels of entry in the vehicle controls in Fig. 3A and 3B?)

*We thank the reviewers for this comment. The viral inputs of HIV-1_JR-FL_ and HIV-1_VSV-G_ were not normalized via p24 and we understand therefore that direct comparisons cannot be made between HIV-1_JR-FL_ and HIV-1­_VSV-G_ consequently. As addressed above, we therefore have removed this conclusion from the paper and have merely emphasized in the main body of the manuscript that we utilised HIV-1_VSV-G_ to merely answer the question as to whether or not pseudotyping HIV-1 with a different envelope would affect the observed dependency of viral entry on the glycolytic activity of the cell (lines 169-170).*

*As mentioned in the methods, we tittered both HIV-1_JR­FL_ and HIV-1­_VSVG_ such that we would be adding equivalent infectious particles/cell in each experiment (i.e. MOI). Because of this, we would like to stress that the ratio of virus/cell is the same*

*We can provide the raw %entry values in the vehicle samples that are used to normalize the data. Please see attached.*

b. A direct comparison is also needed to test whether there is a stronger effect of 2DG (e.g., 100 mM) on HIV fusion than on HIV infection. (The legend to the resultant figure should also give the raw % entry values for the vehicle controls used for normalization.)

*We thank the reviewer for this comment and believe that we have we have addressed this in the above rebuttal text.*

c. Given the above queries, the authors should provide data from several repeat Fig. 3A/B experiments (at least at 0 and 100 mM 2DG), comparing fusion and infection in the same experiment, and in parallel with Env (from the same strain of HIV)- and VSV-G-pseudotyped HIV particles (normalized for input).

*We thank the reviewer for this comment and believe that we have we have addressed this in the above rebuttal text.*

3. The authors report (e.g., line 167) that "HIV-JR-FL particles enter TZM-bl cells via endocytosis". Most of the reported membrane perturbations (to cholesterol levels and membrane order and tension) caused by 2DG treatment were reported in TZM-BL cells (or analyzed in pre- vs. post-infected TZM-BL cells), but these changes were all measured for the plasma membrane.

*It has been reported in the literature (Miyauchi et al., Cell, 2009, de la Vega et al., Retrovirology 2011) that HIV-1 particles fuse within endosomes in TZM-bl cells. Nevertheless, these experiments were performed with spinoculation protocols (i.e. centrifuging the cells exposed to HIV-1 particles at ~2100G for 20-30 min at 10-20 ^0^C to block endocytosis). We should have clarified this point as we (Jones et al., 2017) and others (O’Doherty et al., 2000, J Virol; Guo et al., 2011 J Virol) have studied the effect of spinoculation in the point of HIV-1 fusion and how this alters host biology, specifically the actin cytoskeleton regulation and dynamics, crucial for HIV entry and how this infection protocol will bias HIV-1 entry to endosomes (Padilla-Parra and Dustin., 2016 Trends Mol Med).*

*To avoid this bias and to enable HIV-1 fusion in the plasma membrane, we avoided spinoculation in our infection and single particle tracking protocols. We found that even if endosomal fusion was still possible, when adding the HIV-1 pseudoviruses on the top of TZM-bl cells, we could detect many plasma membrane fusion events. As discussed in Jones et al., 2017 Cell Reports, we found that spinoculation affects the actin cytoskeleton and endocytosis. We have produced now a new figure with new data showing the statistcs for spun and non-spun cells including all possibilities: HIV-1 Endosomal fusion, Hemifusion and Plasma Membrane (PM) Fusion. Only in the case of non-spun cells PM fusion was observed (New Supplemental Figure 4 (Related to Figure 3). We have also produced movies for all examples. Therefore, we were confident that the HIV-1_JR-FL_ entry events that we tracked were indicative of HIV-1_JR-FL_ entry at the plasma membrane.*

*All our single virus tracking experiments in the current work were performed without spinoculation protocols and this should have been stressed, our previous work in that regard also should be cited. We include new data now and cite our paper (Jones et al., 2017) in the text and material and methods (Lines 191-196).*

a. The authors should explain how they propose the properties of the plasma membrane listed above would affect a membrane fusion event that occurs in endosomes.

*As previously explained, we focus on HIV fusion events that explicitly occurred in the plasma membrane as no spinoculation protocols were performed. We now provide at least 12 events in which PM fusion does not progress from hemifusion when cells are treated with 2DG.*

*In regards to endosomes, we are thoroughly interested in and have attempted to measure both membrane order and membrane tension for our pseudoparticles in entering in endosomes. Unfortunately, our MSS tension probe (a gift from Bo Liu) was engineered to only label local plasma membrane tensions as it is engineered to be directed specifically to the plasma membrane. In addition, the Flipper probe, which measures membrane order, only localizes to the plasma membrane (although, there is evidence to suggest that it can be endocytosed, Colom et al 2018). Nevertheless, even if these probes were to be endocytosed in these cells, we believe it would be very difficult to resolve changes in lifetimes with current technology, as the chemical and biological environment of endosomes may change the physics of these sensitive probes used for lifetime imaging, such that any results taken from these regions of interest would be uninterpretable. Furthermore, we believe that endosomes labelled by either probe would possess insufficient photons to gather enough counts for TCSPC FLIM to create lifetime values with reliable statistics. Thus, we are restricted to studying the plasma membrane.*

*We have endeavoured to circumvent this problem by analysing filipin-stained endosomes (identified by Rab5) in TZM-bl cells treated with 2-DG for the same amount of time in the text and have discovered that quite similar to the plasma membrane, there are reductions in endosomal cholesterol content. This data is currently not shown, however, as our goal of the manuscript is to emphasize plasma membrane events. However, if necessary, we are willing to provide this upon request.*

*We have updated our discussion to include these comments to guide the reviewer as to how to interpret our results in the plasma membrane, and that caution should be provided when using this data to determine if similar effects could occur in endosomes (lines 207-215; lines 354-366.)*

b. Or, do the data and conclusions refer to the minor population of HIV particles that enter TZM-BL cells at the plasma membrane? If so, are the plasma membrane effects of 2DG treatment the major reason for the observed inhibition of infection? (Others have argued for a post-entry need for high metabolic activity for successful HIV infection of T cells.)

*Indeed, we refer to the population of HIV particles that enter TZM-bl cells in the plasma membrane (as explained above). We show the statistics for these in the context of spun and non-spun cells and the comparison between endosomal and PM fusion (see above) new Supplemental Figure 4 (Related to Figure 3)*

a. To help address this point, the authors should provide a summary analysis (with statistics) of all fusion phenotypes observed +/- 2DG (binding to plasma membrane but no fusion, hemifusion, full fusion), including whether each type of fusion seen (under each condition) occurs at the plasma membrane or in an endosome (as well as corresponding data for cholesterol-rescued 2DG-treated cells).

*We thank the reviewers for this suggestion. We have now done so (Supplemental Figure 4 (Related to Figure 3)) and clarified this crucial aspect of our research. We have also provided the corresponding movies for each condition.*

4. The authors show that oligomycin does not impact on the level of fusion in MT4 T cells. However, primary T cells have been found to be exquisitely sensitive to oligomycin, with very low levels inhibiting T cell proliferation and activation (Chang et al., Cell, 2013). The absence of impact in the present study is likely due to the "pre-activated" state of an MT4 cell. It will therefore be important to assess whether oligomycin impacts on MT4 metabolism (ATP:ADP ratio, lactate). Differences with the importance of glutamine/oxphos in primary T cells should be discussed.

*We agree that it will be important to assess whether oligomycin impacts MT4 metabolism in the face of acute oligomycin treatment. As such, we have provided 2-photon FLIM data to illustrate that the acute treatment of MT4 cells with oligomycin fails to illicit a change in the redox ratio (i.e. NAD(P)/NAD(P)H) in MT4 cells (Supplemental Figure 1H). Therefore, it seems that as the reviewer has mentioned, the pre-activation state of the MT4 cell has negated the inhibitory affect of the oligomycin. This note is now discussed in the discussion (lines 342-343), keeping in mind the previously published data provided by Chang et al. 2013 to note that this is a stipulation worth noting in our study.*

*Nevertheless, we would like to stress here that this lack of change in metabolic state during oligomycin treatment is not unexpected in the context of HIV-1 infection and supports our hypothesis that glycolytic flux is critical in determining HIV-1 infection. Naïve T cells, which are heavily reliant on oxidative phosphorylation, are highly resistant to HIV-1 infection. It has been previously illustrated that HIV-1 susceptibility increases in differentiated cell subsets (Roederer et al 1997, Schnittman 1990), that T cell activation renders them susceptible (Pan et al 2013, Stevenson et al 1990) and that HIV has adapted to infect CD4 T cells with a specific priming (Cleret-Buhot 2015). Consequently, HIV-1 susceptible MT4 cells are more likely to be in a post-activation state and therefore less susceptible to oligomycin, yet more susceptible to HIV-1 infection (Valle-Casuso, 2018). This has been previously shown in MT4 cells, in addition to Jurkat and CEM cell lines (Makowska, Zabłocki, and Duszyński 2000). Our data enforces this notion therefore that glycolytic flux is what drives HIV-1 infection and that oxidative phosphorylation plays little role. We will further expand on this in the discussion of the text (lines 342-355) to provide context for the reader. We will also provide a sentence or two about glutamine metabolism, although this was not assessed in this study, as glutamine may often be directly involved in T cell differentiation and function (lines 355-359).*

5. The authors find that exogenous cholesterol restores fusion in 2-DG-treated cells. Does it change the metabolic sate of the cell (i.e. ATP:ADP and/or lactate)? As a related question, if the MT4 cells are not sensitive to oligomycin (i.e not dependent on oxidative phosphorylation), what pathways are responsible for a metabolic activity that supports HIV-1 fusion in 2-DG/cholesterol-treated cells? Are cells treated with oligomycin, 2-DG and cholesterol susceptible to fusion?

*We thank the reviewers for this comment. Although we believe that the addition of exogenous cholesterol will not alter the metabolic state of the cell as the cholesterol used in this study was MBCD-conjugated water-soluble cholesterol, we will perform the experiment as requested, utilizing the FRET-FLIM approach previously utilized to answer this question.*

*As argued by the entire paper, we believe that glycolysis is paramount in the early stages of HIV-1 infection, particularly at the level of HIV-1 fusion. The lack of cholesterol in 2-DG treated cells is what drives its inhibition and furthermore, its replenishment in the membranes rescues this fusion deficiency. We can inhibit the formation of cholesterol with an inhibitor to ACoA carboxylase to see if similar affects on HIV-1 infection occur.*

*In regards to the reviewer’s final comment in this section, we believe our data has answered this question already. Treatment with oligomycin not only minimally impacts metabolism in our cells studied, but minimally impacts HIV-1 fusion. Therefore, we believe a similar situation to Figure 4C shall arise.*

**Other specific Issues**

1. General: It is distracting to refer constantly to Supplemental Data when making major points. For example, the multiple paragraphs describing FliptR and MSS probe activity refer entirely to Supplemental Fig. 5 and not to any primary figure (lines 210-242). It would be helpful to make major points in a primary figure and reserve supplemental figures for additional controls or data that cannot be presented in the figures.

*We thank the reviewer for this comment. However, the figures are organized in such a way such that the supplemental figures provide background to the techniques utilized in the study (i.e. Figure 1 and 5) such that they are appropriate for the supplemental. Due to the fact we wish to provide explanation for these figures to guide the reader through the study, it makes sense that we devote substantial amounts of the text to them. We apologise that this is not the reviewer’s preference, however, we shall keep these as current.*

2. Line 53: define Ld domains at the first instance

(Corrected)

3. Line 106: Clarify: What is the "or" option?

(Corrected)

4. Fig. 1A/B: It is suggested to include the HIV data (currently in Supplemental Fig. 1), in addition to the provided data with VSV-G-pseudotyped HIV.

*We appreciate the suggestion, however, for the sake of simplicity, we shall keep it where it is currently.*

5. Fig. 1D images. As an aid to the viewer, outline all infected cells in the corresponding FLIM images

*We thank the reviewer for this comment. We think this is a great suggestion to help avoid any ambiguity and will hopefully clear up point 6 and 7 as well (below).*

6. Figure 1D, Top row-The panels are said to show that cells with higher lactate are infected, so this panel of cells presumably is the same one imaged by two different techniques. However, the suggested correlation doesn't obviously hold in this example. Many of the infected cells in the left panel show no signal on the FLIM panel, including the cells with the highest levels of X gal. Why is this not consistent? Also the alignment of cells between the X-gal and FLIM panel is unclear- perhaps the panels are not identically sized and oriented.
Figure 1D, bottom row: Are the cells on the left represented on the right (FLIM) panel? The images seem unrelated, with different shapes and positions of cells. Again, this example doesn't seem to illustrate the principle of the assay as intended for dual imaging of identical cells.

*We thank the reviewer for this comment and would like to stress that this can be simply explained. As explained in the methods section of the paper, these cells are transiently transfected with these biosensors (i.e. Perceval and Lactate). Therefore, a substantial proportion of these cells will fail to express these reporters and subsequently will fail to fluoresce and consequently fail to have a FLIM signal. We will like to also emphasize that the cells stained by Xgal and the corresponding FLIM panels are from the same region of interest. However, as stated in the methods, we had to come back 48 hours after infection to the same set of cells, so naturally they may have moved slightly. We have tried to be as consistent as possible in terms of the region of interest. Hopefully highlighting the cells will help here.*

7. Figure 2B, 2C. While the statistics show an overlap of BlaM activity and FLIM signal indicating fusion occurs in the lactate-high cells, the individual panels are not showing the same cells. The boxed areas in the top panels (BlaM) are not the same cells as the FLIM signals in the bottom. Since the entire premise is that both signals are measured in the same cells, the investigators should show both signals in the same panel of cells and not in distinct wells or populations. The same issue occurs with Fig. 2C. It is unclear why distinct cells are being shown if the point is to measure both signals simultaneously at the single cell level.

*We thank the reviewer and would like to stress that this can be simply explained by the same comment as above. These are the same cells. As mentioned in our response to minor comment #5, we will circle the cells we are referring to in order to avoid any ambiguity. However, we must emphasize here that these cells (i.e. in the FLIM and in the Xgal images) are the same cells.*

8. Fig. 3C would benefit from showing direct comparative data for particle fusion in untreated vs. 2DG treated cells.

*We thank the reviewers for this comment. As highlighted in the third major comment, we will provide the additional images and videos as requested to help assist the manuscript in conveying our message that 2-DG inhibits plasma membrane fusion in TZM-bl cells, and that JR-FL may fuse with TZM-bl cells at the plasma membrane, in addition to endosomes.*

The single-particle tracking experiments presented in Fig. 3C suggesting hemifusion following 2-DG treatment are not convincing as presented. An example of loss of DiD signal is shown in the bottom panel of 3C, but the part of the figure stated in the Figure legend as double-labelled particles losing eGFP signal seems inadequate. Instead there is a line tracing of loss of DiD signal in this position of Figure 3C. In any case, a quantitation of events is needed comparing untreated vs. 2-DG-treated cells in order to conclude that 2-DG has arrested fusion at the hemifusion stage. Movies should be provided in supplemental data to establish the technique and illustrate the hemifusion vs. endosomal fusion events that the authors refer to.

*We hope providing another panel for JRFL fusion is sufficient here. We can go ahead as well and provide a movie with these as well in the supplemental figures.*

*9. Numbers of independent experiments that were performed are not always indicated (e.g., Figures 3, 4).*

*We thank the reviewers for this comment and have updated all figure legends with the number of experiments and cell lines utilized in each experiment.*

10. Lines 831-833: "Virions entering via plasma membrane fusion will have their DiD signal infinitely diluted in the plasma membrane whereas the eGFP-gag signal is retained and mobile". Is that always the case with fusion at the plasma membrane? Why wouldn't GFP-gag enter the cytoplasm and disassemble (i.e. lose GFP signal) following fusion at the plasma membrane?

*We thank the reviewer for this comment and suggest that the reviewers read de la Vega et al Retrovirology (2011). An exquisite overview of the SVT method used is located in this paper. Yes, in general and for the length of our acquisitions, fusion at the plasma membrane with HIV-1 pseudoviruses will create a gag particle signal that is mobile and needs approximately 30 minutes to disassemble, which has been previously shown by others (Mamede et al. 2017, PNAS). Given that our length of acquisition was meant to scrutinize viral particle entry and not uncoating, our imaging was restricted to mostly 10-15 minute acquisitions. Therefore, there will always be a time delay between dilution of the DiD signal followed by an eventual loss of eGFP, but the time difference between these two events will be substantial. Consequently, one can successfully distinguish between PM and endosomal entry. We have updated the text in lines 831-833 to prevent any ambiguity in this field and to enhance understanding. And also provided a new supplementary figure showing how spinoculation can alter the point of fusion Supplemental Figure 4 (Related to Figure 3)*

11. Line 905 is confusing: "2DG-treated conditions (i.e., no 2DG)".

*Corrected*

12. Add to all Fig legends (where the information is currently not provided), what cell type was used: TZM-Bl or MT4. The two cells show some significant differences, e.g. effect of 100 mM DG on cholesterol levels (Fig. 4B): ~50% inhibition for TZM-BL vs. ~20% inhibition for MT4.

*We thank the reviewers for this comment and have updated all figure legends with the number of experiments and cell lines utilized in each experiment.*

13. Statistical analyses are needed (where they are not currently provided) for all bar graphs. Some effects are small (e.g., effect 100 mM 2DG on cholesterol levels in MT4 cells [Fig. 4B, bottom and Supplemental Fig. 4A.

*We thank the reviewer for this comment and have appropriately provided all statistical analyses as requested.*

14. Lines 848-851: Should this text be in the legend to Fig. 4B-Supplement?

*We thank the reviewer for this comment, however it should be as written.*

15. Line 197: State which concentration of MBCD (i.e. 1 mM) caused a similar decrease in plasma membrane cholesterol as 100 mM 2DG. (From Supplemental Fig. 4A, 5 and 10 mM MBCD caused much stronger decreases).

Corrected

16. Fig. 4C, bottom images: Why are the cholesterol values now given as mM? Previously they were given as micrograms (presumably micrograms/ml). i.e., how do the values for cholesterol in the bar graph (Fig. 4C, top) compare with those listed above the images in Fig. 4C, bottom?

*We thank the reviewer for this comment and would like to emphasize here that this is a type and has now been replaced. It should read 100 µg/mL, 200 µg/mL and 400µg/mL, as described in the text.*

17. Fig. 4C, bottom right: Why is there less fusion in 2DG-treated cells supplemented with 20 mM cholesterol than in 2DG-treated cells supplemented with 5 mM or 10 mM cholesterol?

*I think this image was chosen by mistake…We can replace with the correct image.*

18. Lines 200-201 refer to filipin fluorescence images in Supplemental Fig. 4C. However, there is no Fig. 4C.

*We thank the reviewer for this comment. We have corrected the text.*

19. Lines 205-206: It would be more informative to say ~80% recovery at 100 ug/ml cholesterol.

*We thank the reviewer for this comment, however, we have kept the text accordingly.*

20. Line 208: There is no Fig. 4D.

*We thank the reviewer for this comment. This should have read “Figure 4C, bottom row”. Please accept this correction.*

21. Lines 221-222 reads: "Treatment with MBCD showed reductions in lifetime by over 200ps at 1mM concentrations (Figure 5a, 5b)". There are no depictions for MBCD-treated cells in Fig. 5.

*This was referring to supplemental figure 5a. We’ve corrected this accordingly.*

22. Lines 252-291: The authors refer here to Fig. 6. Should the references be to Fig. 5?

*We thank the reviewer for this comment. Yes, thank you for the correction. It has been corrected.*

23. Line 840 gives p value notations for Fig. 3. Provide corresponding asterisks in Fig. 3.

*We thank the reviewer for this comment. This has been resolved.*
24. Line 857 gives p value notations for Fig. 5A. Provide corresponding asterisks in Fig. 5A.

*We thank the reviewer for this comment. This has been resolved*.
25. Lines 865-66 give p value notations for Fig. 5B. Provide corresponding asterisks in Fig. 5B.

*We thank the reviewer for this comment. This has been resolved.*

26. Lines 315-320: Modify based on new experiments on relative sensitivity of HIV Env- and VSV-G-mediated fusion/entry to 2DG treatment (as per Major Comment 1).

*We thank the reviewer for this comment and have removed any notion of comparison between VSV-G and JR-FL in these lines in the prior version of the manuscript. We have now replaced this with general comments on that the arrest of viral fusion by 2-DG treatment is not unique to HIV-1_JRFL­_. Please accept these comments in conjunction with our response to Major Comment 2.*

27. In the presence of 2-DG, fusion decreases from "100" to "20" but in the absence of glucose, it decreases to "60". How do the authors explain this difference?

*We thank our reviewers for this point and recognize that we should have briefly mention the discrepancy in the body of the paper, which we now have added in lines 170-173. We mimicked glucose starvation by washing cells with PBS x 3 after cells were in DMEM_complete_. After this wash, we incubated these cells in glucose-free DMEM to mimic glucose starvation for an acute period (2 hours). We believe that glucose still intracellular, as well as other glycolytic intermediates present at the initial time of starvation enabled glycolysis to minimally continue during the starvation process, which could result in an arrest in fusion that did not quite reach the level of 2-DG treatment. We do have data where cells were starved for longer periods of time (i.e. up to 12 hours). However, in addition to keeping with the acute nature of glycolytic arrest emphasized in the paper, cell death at this point increased beyond 20% of the population and we decided to not include this data consequently.*

28. In order to show the importance of glycolysis in the cholesterol content of primary CD4 T cells, the authors can perform a CD3/CD28-stimulation of CD4 T cells (for 2-3 days), add 2-DG for a short time period, and then assess cholesterol levels.

*We thank the reviewers for this comment and will perform the experiment as suggested. We will respond with the corresponding data from this experiment in due course.*
