# Supplementary material for: Single-cell glycolytic activity regulates membrane tension and HIV-1 fusion
Source: PLoS Pathog. 2020 Feb 21;16(2):e1008359. doi: 10.1371/journal.ppat.1008359 (PMC7055913; doi:10.1371/journal.ppat.1008359)
Supplement: Attachment — Submitted filename: Rebuttal Coomer, Carlon et al 2020_Final.docx [file ppat.1008359.s009.docx]

*Below our point by point response to all reviewers (in italic):*

**Part I - Summary**
Please use this section to discuss strengths/weaknesses of study, novelty/significance, general execution and scholarship.


Reviewer #1: This reviewer was looking forward to evaluating this manuscript as corresponding author usually does very nice work. However, this reviewer feels that current manuscript has many claims that are not fully supported by data presented. The major claim (for this reviewer) being the linkages amongst: (i) glycolytic activity; (ii) membrane tension; and (iii) HIV fusion.

It is my opinion that authors have used a sub-optimal system to link the HIV biology with glycolytic activity or membrane tension. Incorporation with relevant T cells data on key aspects would have provided huge amount of reassurance.

*We appreciate this comment and in this revision have included data with primary T cells. More detailed information is given below.*

In terms of linkage between glyolytic activity and membrane tension, the interesting part begins when FliptR dye is used, BUT the data appear to fell short on definitively making a strong link between glycolytic activity [metabolism] and membrane tension (at least for this reviewer).

*We agree with the reviewer. We have now improved the FliptR figure with new Images and put it as a main Figure 5 in the text. These data together with the MSS tension FRET biosensor (New Figure 6 and 7) establishes a strong link between glycolytic activity, HIV fusion and membrane tension.*

There is large amount of errors within the manuscript (in particular with figure and supplement figure referrals), which unfortunately make this assessor has (perhaps unnecessary) doubt on some of the work.

*We apologize for the errors present throughout the manuscript and agree with the reviewer. We have now, with the help of the reviewers, corrected these errors and improved significantly the manuscript.*

Overall, authors have used a lot of cool and exciting techniques in this paper, BUT the authors seem to not have careful considered the limitations of various technique used (at least this is the interpretation of this reviewer). Claims being made in the paper are NOT readily justified in my view. There are fair bit contradictions presented in this revised manuscript that I evaluated (see below).

*Thanks for this comment. We have employed advanced imaging because we are convinced that these approaches are the best suited to test the hypotheses of our study.*

Overall, authors try to look at the impairment of metabolic effect might have on virus entry. Authors claims that there is a link between metabolic state of the target cell and the ability for the virus to infect. If one takes a simple step back, such premise is not surprising at all. Virus (by definition) hijack host cell system to achieve its own gain. From the point of virus entry, establishment of infection, genetic materials and protein productions, to the stage of assembly of virus, ALL rely on the host cell to be somewhat healthy to begin with - ie metabolic active to generate energy to the rest of the cells, thereby invading virus can take advantage of.
On the other hand, if authors are trying to show a particular subset of metabolic events is purposely hijacked or manipulated by viruses, it would be extremely interesting. To do that, one should demonstrate any observed effect is related to such subset of metabolic events, but the manipulation was NOT affecting the global process (issue of pleiotropic effects).
Unfortunately, in this manuscript, authors have chosen to use a very blunt instruments (in terms of biological systems [TZM-bl] as imaging tools are top notch) to demonstrate this point. Authors appear to over-push the imaging technology fronts that this lab is good at, without carefully consider all the limitations (at least this is this assessor's opinion). Please see specific comments below.

*We acknowledge this instance and have now performed fusion experiments with TZM-bl cells, MT4 T cells and also primary T cells. All these experiments (explained in the text and below) clearly show the impact of glycolysis on PM cholesterol and its impact on HIV-1fusion.*

Having said that, all of these imaging are state-of-the-art techniques. Some time it is less clear for this reviewer whether authors are show-casing these techniques or answering a specific question for this retrovirus.

*We re-iterate this vision throughout our rebuttal: the imaging approaches employed here were chosen as the best approach possible since they allow us to increase the resolution to assess HIV-1 fusion to single cells and single viruses in different metabolic contexts. Without being able to have this resolution, the events described in this manuscript would not be appreciated. This is a key idea: advanced imaging approaches allowed us to improve the resolution of HIV-1 fusion from bulk to single viruses so that we could find the inter-relationship between metabolism, membrane order and tension and HIV-1 fusion.*

Key points in summary include the following:

2DG impairing glycolysis, then affect lipid membrane function in association of cholesterol. This could simply be a reflection of a dynamic process in lipid membrane (that requires energy) being important for virus entry?
My own interpretation is that affecting glycolysis could simply be that target cell for infection require the membrane to be healthy (to have dynamic lipid movement) to enable virus to enter. in that sense, if the majority of metabolic process are in idle, it might not be surprising for authors to see these outcomes.

*We argue this vision with specific comments related to experiments below. It is worth mentioning, though, “healthy membrane” is a rather vague term. We, in this paper, are not dealing with trivial issues related to the reviewer’s claim almost equivalent to suboptimal cell culture conditions, but specific perturbations that are within the range of healthy cells in vivo. Importantly, our methods are able to probe these specific perturbations in membrane structure and composition in specific terms; always within the broad range of what a “healthy membrane” means and its parameter space.*

cholesterol-lowering medication in HIV infection is a pretty big stretch by the authors as far as this reviewer is concerned.

*Although our new experiments showed similar reduction in HIV-1 infection upon treatment with cholesterol lowering drug Simvastatin, we agree with the reviewer and have erased these lines from the manuscript. Even if we have found the relationship between cholesterol concentration in the PM and HIV-1 fusion this remains to be tested and is out of the scope of this work.*

From author summary, ‘regulating cholesterol’ was mentioned. It might not be related to cholesterol regulation on plasma membrane. It is more likely related to maintain dynamic movement of plasma membrane. Observed cholesterol supplement effect could be secondary effect of cholesterol uptake that permits / restores a more dynamic movement of plasma membrane under these 'stress' conditions.

*We thank the reviewer for this comment. Indeed, a dynamic membrane is required for supporting entry of virus into the host cell. Our data supports this view. However, we observed dynamic fluctuations in membrane tension during virus entry even in the presence of 2-DG treatment in figures 6-7. Furthermore, infinite dilution of the fluorescent DiD lipids in the plasma membrane was observed during our single virus tracking experiments. These results altogether demonstrate dynamic movement of lipids in treated and untreated cells. We should not assume that membrane dynamics is the sole factor playing a role in the blockage of virus entry. With the abundance of cholesterol data (i.e. filipin, fliptR), we are drawn to the conclusion that 2-DG treatment disrupts cholesterol availability at the plasma membrane and also within endosomes.*

This assessor has different interpretation from authors on their comments on metabolism, immunity, and viral infection. Authors cited Buck 2017 and Pallett 2019, and I have gone in to read those papers. My personal read is that metabolism needs to be active to allow cell to perform. Likewise, if a cell is dormant and is not doing a lot, it will not be any good to serve immune function to counteract pathogens. Similarly, a less active cell will not be effective for pathogens to infect that lead to production of progeny pathogens. This is different from a sick cell that can still produce energy source, but innate immunity is down. A key issue perhaps is how much the host cells are in idle (80% functional vs 20% functional in energy production). This relationship is particular important for viruses, as viruses rely on host cell machineries to achieve its own gain. Unless the target cells and host cells are 'fairly' active in generating resource for pathogens to take advantage of, there is not a lot of use for viral pathogens. I think we all agree that biological process requires energy in one form or the other.

*We think that the validity of the work presented in the manuscript is not in contradiction with these ideas. Further discussion on metabolism is found below (next chapter Part II: Major Issues).*

The entire infection process requires energy, and viruses DO NOT bring in its own energy production source. The simple explanation (that authors observed) is an effect at the entry stage of virus-cell interaction, which reflects the very first step of energy expenditure process that virus relies on the host cells - ie entry.
Authors described hemi-fusion event and membrane curvature in an attempt to link cholesterol in HIV infection. However, the system that the authors used also including the VSV-G HIV system, which is unrelated to HIV Env. VSV-G mediated endocytosis entry is largely unaffected by cholesterol (at least with particle-associated cholesterol, and I recalled at least 3 labs have independently reported this). On that note, making it more difficult for the authors to link their story with cholesterol specifically. Again, to me, the phenotype is more likely a general impact on metabolism. More concerns about hemifusion events are described below.

*We have now clarified our real-time single virus tracking experiments and clearly established that cholesterol depletion affects HIV-1 fusion at the point of hemifusion. (see more details in next chapter Part II: Major Issues).*


With PLoS Pathogens, there is NO limit on numbers of figures or word count. There are many supplement figures in this paper should be incorporated as main figures. Authors should correct this before publication. Authors claim current format is intended to be helpful for readers, and I do not share their enthusiasms. It looks though other reviewer is feeling the same. Current formats appear to be something originally submitted to another journal (eLife for example), and this format is not helpful in my opinion. in fact, there are A LOT of errors within this current manuscript that is very damaging to the paper (see specific below).

*We agree with the reviewer and accept this criticism. We have now changed the format, we have included important figures as main figures (e.g. FliptR, Fig. 5 and MSS Fig. 6), included new experiments (Supplementary Figure 2A, 3, 4C-E) and figures with primary cells and increased the quality of all Figures (both main and supplementary figures),*

**Reviewer #2**: Coomer et al. report a beautiful study of the importance of the properties of the target membrane on HIV fusion, and its link to the metabolic state of the cell. They find that reduction in glycolysis removes cholesterol from the plasma membrane, which increases membrane tension. This increase in tension is less favorable for HIV fusion. As a result, virions entering at the plasma membrane stall at the hemifusion stage, as evidenced by observation of lipid mixing in the absence of content release. While the importance of target cell metabolic state and the physical properties of the membrane have previously been reported as important to HIV fusion, this study makes clear the inter-relatedness of these previous observations and generates a more unified understanding.

As one has come to expect from the Padilla-Parra laboratory, the authors use quantitative optical microscopy approaches, and a variety of fluorescence-based assays, which appear to be rigorous and well executed. By my evaluation, the previous reviewers were appropriately thorough; and the authors have nicely addressed all major points raised. I therefore have only minor comments.

**Reviewer #3**: Overall, I thought this was an interesting paper. I think the assays that the authors have used are clever and allow them to begin to answer very difficult questions in our field. I particularly liked the coupling of the FLIM images with infectivity assays and fusion reporters in non-treated cells, since as the authors mentioned, the experiments are internally controlled and the metabolic pathways of the cells are not perturbed. At times, I wish the data they presented were a bit cleaner, but the statistics look good. The biggest frustration I had with this paper was the number of errors in labeling and writing. These errors made important parts of the paper very difficult to decipher, and at times, I was left wondering if I interpreted their errors correctly and was understanding the data properly. To be honest, I am not someone who often picks up on these types of errors, so the fact that I found so many suggests that it’s a significant problem. The authors need to read this paper VERY carefully and correct all errors.

*We thank Reviewers #2 and #3 and apologize for the amount of errors in the previous manuscript. We have endeavored to improve the manuscript following all suggestions from the three reviewers and our own criticism. We feel that the manuscript has pretty much improved thanks to these comments and our own criticism.*

**Part II – Major Issues: Key Experiments Required for Acceptance**
Please use this section to detail the key new experiments or modifications of existing experiments that should be absolutely required to validate study conclusions.

Generally, there should be no more than 3 such required experiments or major modifications for a "Major Revision" recommendation. If more than 3 experiments are necessary to validate the study conclusions, then you are encouraged to recommend "Reject".


Reviewer #1: Figure 1 TZM-bl. TZMB1 spreads out very well, and they are excellent for cell morphology in terms of imaging. TZM-bl are also very tough-resilient, and they are not real cells for HIV infection. MT4 is good, but still limited. For the claim to be linked with HIV, it would be necessary for author to repeat figure 1A with PBMCs (2-DG dose response) along with cell viability. Maximum impact is 50% reduction of infectivity with VSV-G pseudo typed HIV. I wonder what the impact it would be using PBMCs and R5-tropic (or even transmission strain HIV) to infect. The key concern is that the 2-DG treatment described was way too tough to the host cell to begin with. By impairing the major metabolic processes to the host cells, these cells are essentially too weak (perhaps even dying) to do anything, therefore not able to support HIV infection as consequence. If authors insist on their claim with HIV, I think it is only fair that they have appropriate T cell infection data.

*We thank the reviewer for his/her comments and appreciate the limitations regarding the utilization of TZM-bl cells. We have extended our research in primary CD4 T cells as far as we were experimentally capable. We kindly would like to refer the reviewer to the provided Rebuttal Figure 1 (below) and comments below (in response to the reviewer’s comments made in regards to Figure 2) for further explanation.*

*We have performed experiments to assess fusion of HIV-1 VLPs in primary T cells treated with various concentrations of 2-DG and administration of simvastatin (please see Supplemental Figure 3). These results illustrate that even primary T cells treated acutely either with 2-DG or simvastatin are less susceptible to fusion by HIV-1 HXB2. In addition, we have performed filipin staining in primary T cells treated with various concentrations of 2-DG (Supplemental Figure 6). These results directly illustrate a recapitulation of our results demonstrated in TZM-bl cells, specifically that cells treated acutely with 2-DG are depleted in cholesterol.*

*In regards to the utilization of 100mM 2-DG in this study, we kindly would like to refer the reviewer to the publications cited in the paper, particularly the most recently published study (Valle-Casuso 2019) which utilized 100mM 2-DG for at multiple days, and even up to two weeks on PBMCs and did not even achieve 50% cell death. In this study, we used 2-DG for merely two hours, less than 1% of the time used in the previous study. In this context, and supported by our propidium iodide staining, we would like to directly refute this comment stating that this is “too tough” of a treatment for the cells utilized.*

On the other hand, if authors were changing their claims, experiments in T cells might not be needed. More specifically, the beautiful imaging data are excellent. To me, they demonstrate to me that energy requirement is needed to have a dynamic membrane (including cholesterol movement). Such dynamic movement of plasma membrane as well as membrane related endocytosis process are fundamental for particles uptakes. What authors are seeing in both HIV particles in TZMBL and VSV-G HIV in TZMBL are reflection of that. This could well be a general principle for particles uptake (including many viruses and perhaps even exosomes). Such general principle could be best demonstrated using various viral envelope pseudotyped particles (Zika Env, SARS Env, Ebola Env and VSV-G to just named a few) plus a number of other relatively easy to image fibroblast typed cells. Authors can even use the exact same imaging system that they have right now.

*We thank the reviewer for this discussion comment and believe that he/she offers an interesting point. Indeed, a dynamic membrane, including the movement of cholesterol, would be required for supporting entry of virus into the host cell. Our data, in part, supports this hypothesis. However, particularly in (new) Figure 7, we still see dynamic fluctuations in membrane tension during virus entry even in the face of 2-DG treatment. Furthermore, infinite dilution of the fluorescent DiD molecules occur in the plasma membrane during our single virus tracking studies, demonstrating the dynamic movement of lipids. Hence, we cannot assume that membrane dynamics is the sole factor playing a role in the disruption of virus entry. With the abundance of cholesterol data (i.e. filipin, fliptR), we are drawn to the conclusion that 2-DG treatment disrupts cholesterol availability at the plasma membrane and in endosomes.*

Legends in Figure supplement Figure 1A are not adequate. Which one is HIV-1(VSV-G) or HIV-1(NL4-3)? Is VSV-G on top of NL4-3 or VSV-G on top of Envelope negative HIV? In fact, the presentation of supplement Figure 1A is not up to usual standard of this team. I would imagine authors should synchronise all fonts and font size throughout all figures (including supplement) to deliver their points to reviewers in order to help to demonstrate their case effectively, as well as best to illustrate the quality of works being done. Presentation of supplement figures are no less important from the main figures.

*We thank the reviewer for these comments and have addressed all comments from all reviewers concerning the suboptimal construction of the figures. We hope that the reviewers are now satisfied with the figures and supplemental figures in the revised version of the manuscript.*

With Figure 1 supplement figure 1B, is that laconic or perceval? Similar question with supplement figure 1d. what is on the label with this supplement figures vs what is written in the supplement figure legends are completely opposite - which one is the truth? Same question for Supp Fig1E vs Supp Fig1F. Noticed author rebuttal, but I am not sure error has been corrected - at least with the version I have.

*We thank the reviewers for this comment and apologize for the confusion this supplemental figure has caused. Indeed, the figure legend in the supplemental figure had been mistakenly swapped, particularly in the figure legend for supplemental figure 1B and 1D. We hope that the reviewer can appreciate that this has now been corrected. We have now created an updated supplemental figure 1 and corresponding legend to correctly label the panels appropriately.*

Authors should have better explain what the oligomycin control is used for, and what that meant. Currently, it is very inadequate in this assessor's view.

*We thank the reviewer for this comment and have added short explanation in the manuscript in line 140-147.*

With the schematic on Fig 1C and the ACTUAL data in Fig1 Suppl Fig1F-H, authors seem to suggest the metabolic state of the cell are significantly affected by 2-DG. With this in mind, would it not true a simple explanation being that the 2-DG treated cells are now so weak to produce energy source that virus can no longer hijack the cellular machineries to establish infection? A trivial and simple explanation. An important character of virus being that it relies on host cell to replicate, and the key issue for that being virus cannot generate its own energy source and metabolically inert.

*We thank the reviewer for this interesting comment and would also like to take this opportunity to offer guidance to the claims made above and further below by the reviewer in regards to 2-DG and cellular function. We appreciate the reviewer’s claim that 2-DG could prevent the hijacking of cellular machinery to prevent productive infection and viral replication. However, we would like to stress the following points:*

- *We are not investigating the effect of 2-DG on productive infection or viral replication in this manuscript. We are clearly, and we have emphasized this point, assessing the effect of glycolytic activity on virus entry. Therefore, downstream processes (e.g. integration, viral genome replication, assembly, egress) cannot be assessed by this study, although this point can be discussed by other readers, reviewers or in future works.*
- *We would like to take this opportunity to indicate that multiple studies, including most recently published by Valle-Casuso et al. 2018, have subjected primary T cells with relatively similar concentrations of 2-DG for days, with minimal loss in viability. In the extreme, this paper has also treated cells with similar concentrations of 2-DG for up to two weeks and still achieved appreciable levels of infection.*
- *If we were to hold the reviewer’s comments to be true, then it would be difficult to explain the results in Figure 3B, which illustrates appreciable amounts of virus entry, and Supplemental Figure 1A, where the majority of the MT4 cells treated with a live/dead marker are still viable after treatment with 2-DG, and furthermore, support appreciable amounts of infection.*
- *We would also like to point out that even in the face of glycolytic inhibition, recent publications have illustrated that primary effector CD4 T cell function is preserved in the face of glycolytic inhibition for even up to 3 days, and that these cells are still viable and capable of T cell functions such as IFN gamma production and TNF production (Renner et al 2019, Cell Reports).*

*As this notion by the reviewer has been made multiple times in his/her assessment, we will refer the reviewer to this commentary here to rebuttal any recapitulation by the reviewer below regarding the use of 2-DG on virus replication and productive infection, as this was not the purpose of the manuscript in its current form.*

Arguments made by authors from lines 142 to 152 are not interpretable given the conflict of figure 1 supplement figure 1B-E legends

*We thank the reviewer for this comment and appreciate her/his concern. We hope that now the conflict between the supplemental figure 1 legends with supplemental figure 1 B and D has been resolved (please see above) that the reviewer is now able to appreciate the arguments made in lines 157-170.*

With limitation / conflict of figure 1 supplement figure 1B-E legends, paragraph from line 153 to 161 are not easy to understand

*We thank the reviewer for this comment and appreciate her/his concern. We hope that now the conflict between the supplemental figure 1 legends with supplemental figure 2 has been resolved (please see above) that the reviewer is now able to appreciate the arguments made in lines 157-170.*

At the first glance, data in Fig 2 are really cool set up and experimental approaches. However, the choice of using TZM-bl cells appears to be a waste opportunity for this assessor. Only if authors apply these in more relevant cells. Just because TZM-bl are easy to image, it does not mean that TZM-bl is best for the current biological question in hand (see below with more details or above for tailoring claims against data presented).

*We thank the reviewer for this comment and do agree with the reviewer. We choose TZM-bl cells at first, because it allows to report HIV infection with high sensitivity via B-galactosidase assay., In the manuscript’s current form, we have extended to primary cells as much as we were experimentally able to do. We have attempted the proposed experiment multiple times (please see attached data to this rebuttal, below). As you can see in the attached data set, we have attempted to create RNA from the Perceval and Laconic (not shown) plasmids and subsequently electroporate this synthesized RNA into primary CD4+ T cells. This strategy has been successfully adopted by us before, and indeed control GFP was produced successfully in primary CD4 T cells. We were successful in the creation of scaled-up, stabile, poly-A-tail-containing RNA products from both plasmids. However, at both 24 and 48 hours post-transfection, we were unable to detect any fluorescence (i.e. Perceval expression) in any of the CD4+ T cells. In regards to Laconic, although we were able to successfully detect fluorescence in CD4+ T cells, the lifetimes extracted from the CD4+ T cells expressing Laconic were nonsensical (e.g. 0.8ns—very far from the natural lifetime of YFP), and most likely due to defective protein folding. Despite multiple attempts, we could not acquire proper lifetimes for this protein. Therefore, we have attempted this experiment in the most ideal cells possible. Unfortunately, due to these shortcomings in the primary cells, we are left with the TZM-bl and MT4 2PE data, which is promising and reproducible in three separate experiments.*

*To assist the manuscript in its current form, we have performed multiple BlaM assays with primary CD4 + T cells in the presence / absence of 2-DG to assess the effect of glycolysis inactivation on viral entry in a more relevant cell line. Furthermore, we have additionally performed also experiments with filipin-based cholesterol content quantification in primary T cells treated with 2-DG and have illustrated results that are comparable to the TZM-bl-based data. We hope these experiments assure the reviewer that we have attempted to our best capability that we endeavor to perform experiments in more relevant cell lines.*

With Vpr-b-lam assay, the enzyme b-lam is from virion, substrate CCF2 is passively introduced (does not require metabolic events of the target cells). There are a lot of HIV ‘fusion’ events seen in 2B. What is the background level of b-lam activity with this batch of TZM-bl cells of the authors. TZM-bl or HeLa are well known engineered cells, TZM-bl (and HeLa) can vary from one lab to the next, early day cell engineering often utilise plasmid contain ampicillin resistant gene (blaTEM-1). It would be great (in fact important) for authors to confirm their TZM-bl cells to have low background of b-lam gene products (without HIV infection) to reassure their observations of red (presumed fusion events) are indeed related to HIV entry (therefore releasing b-lam from Vpr-conjugation). This assessor has seen data of different TZMbl used for b-lam entry assay with significant different levels of backgrounds. Authors should clarify this. There is a scale bar in second panel of 2b, but actual scale was not described in the legend.

*We thank the reviewer for this comment and would like to offer guidance on this panel. As explained clearly in Figure 2A, which describes the B-lactamase assay, we do not use the platform described in this review by the reviewer (i.e. the use of a blaTEM-1 gene in the cell line being analyzed). Instead, we utilize an assay where the enzyme is utilized by the virion. We have utilized a No Env control, essentially naked virus particles containing Vpr-Blam, which lack any capability of entering TZM-bls loaded with CCF2, as our background control. We have provided this image for the reviewer for assurance as an attachment to this rebuttal as attachment 2, below. The background provided by these images enabled us to assess whether events captured when utilizing HIV-1_VSV-G_ or HIV-1_JR-FL_ as in order for a fusion event to be identified, the cleaved CCF2 fluorescence intensity must have been higher than this background level, plus two standard deviations, to be considered ‘positive fusion’.*

*In regards to the scale bar in panel 2B, we have made more clear in the figure legend as what the scale bars are referring to in the figures. To be diligent and clear, we have now created space in the figure legends for all figures, which have a corresponding scale bar in the figure.*

With data in Figure 2, authors suggest that low metabolic activities are related to lower fusion events for HIV entry. Here is an alternative interpretation. Assuming that there is background b-lam issue with the TZMBl cells being used, therefore it is possible that lower metabolic activity would lead to less endogenous b-lam being expressed, hence, loading of CCF2 dye will give rise to less portion of red (presumed fusion positive cells), while adding HIV into these cells will lead to smaller fraction in terms of changes to ‘presumed fusion positive states’. Authors could still be correct, but these alternative possibilities must be eliminated. Also, TZM-bl is great for endocytosis mediated entry (which are also supported by data presented by this team). At the end of the day, best possible controls please.

*We thank the reviewer for this comment, however, we would like to emphasize again that the BlaM assay being described by the reviewer is not in accordance with our protocol, and is indeed an entirely different system. We would like to refer the reviewer to the Methods section of the manuscript. In our BlaM assay, which is clearly described in the methods, we do not utilize cells capable of endogenous BlaM expression and therefore the “background b-lam issue” being referenced to by the reviewer is void and not applicable. To be clear, the viruses added were neither produced in cells treated by 2-DG, nor were the cells analyzed capable of expressing endogenous BlaM. After virus addition, the cells were loaded with CCF2 dye, and only then assessed for fusion.*

From the paragraph starting in line 177, authors indicated that treatment of 2-DG does not affect viability of cells based on propidium iodine staining. Two questions emerge: (i) is propidium iodine staining the best assay for this?

*We thank the reviewer for her/his concern in regards to the use of propidium iodine staining in regards to cell viability/death. The utilization of propidium iodine staining was to rule out dead cells in our microscopy-based analyses and to also determine if there was any overwhelming cell death during 2-DG treatment. A cell death assay was also utilized in Valle-Casuso et al 2018 to look at T cell viability after 2-DG treatment. In addition, plenty of papers utilize cell-death assays (i.e. live/dead, PI) to assess cell death post-drug treatment. We assume that this question is linked to the comments made by the reviewer below in regards to cell viability after 2-DG treatment, which we have answered above already extensively in the above section of this rebuttal.*

*Again, we would like to stress the following points. Multiple published studies have utilized similar or identical concentrations of 2-DG in both cell lines and primary T cells for much longer periods of time (i.e. days or weeks) in contrast for the short couple of hours we subjected these cells to this treatment. These studies not only were able to show that cells were capable to support normal T cell function, but also illustrated that multiple days or weeks of 2-DG treatment still enabled appreciable levels of HIV-1 infection (although decreased), in addition to sustainable cell viability.*

*To appease the reviewer, we have performed some additional experiments also in primary T cells, which have been discussed above in detail.*

As described above, what if the cells have become SO fragile / weak that are no longer able to support most of their biological activities due to not making enough ATP (but they are not yet dead). As a consequence, much of the mechanism viruses rely on to infect the host are no longer available. These top down overall effects on shutting down metabolic process would complicate interpretation. In the case the propidium iodine staining, the cells HAVE to be dead in order for the dye to go in, hence it is not fair to say that 2-DG does not have toxic effects to the cells. These cells are likely to be so weak but not dead JUST YET – would you not consider this cytotoxic effect or pleiotropic effect? (I would); (ii) Also, HeLa based cells (including TZMbl) are generally MUCH tougher than many other cell types in the lab, making the thresholds of picking up propidium iodine stains much higher. This reviewer would suggest that parallel experiments should be done on primary T cells to begin with, in particular given authors’ claim on potential clinical application by extrapolating these observations on cholesterol lowing medication in HIV infection (assuming HIV linkage remains a key claim from the reviewers).

*We thank the reviewer for this comment and would like to kindly refer him/her to the above comments for a response to this reviewer’s critique as this comment has been answered previously.*

Another way to look at it would be dynamic movement of lipids (including cholesterol movement in PM) requires energy. What authors have observed (impacts on HIV entry in TZMBl cells) is simply reflection of this defects. In the other word, the phenomenon with HIV entry is a real out of plasma membrane dynamics, but not a SPECIFIC event that is unique to HIV. Perhaps consider showing this as general principle for various particles uptake (including exosomes across larger number of fibroblast cell types? General principle seems to be more important to me than HIV infection.

*We thank the reviewer for this comment and would like to kindly refer him/her to the above response to this reviewer’s critique as this comment has been answered previously. The reviewer is correct that we cannot rule out that this phenomenon may be broadly applicable to multiple types of viruses entering their host cell, the data presented in this publication cannot be simply explained by the reviewer’s brief conclusion that these phenomena is simply restricted to dynamic lipid movement. If this were the case, the fluctuations in PM tension as illustrated in figure 5, and the dilution of DiD dye in the membrane would not be seen. Furthermore, if these principles were solely due to lipid membrane dynamics, such a broad cause would have a similar effect amongst multiple viruses, which is not the case for VSV-G-pseudotyped entry as illustrated in the manuscript. Although we do recognize the reviewer’s point that general principles are valuable, our discussion in its current form is restricted to HIV-1.*

By the way, if authors feel that they are correct that cholesterol lowering drug can impact on HIV replication in a way that they suggest, perhaps they should simply do that experiment using primary cells and physiological relevant dosage of cholesterol lowering drugs to see whether HIV entry is affected based on their hypothesis. If their claim is true, it will be a much stronger paper using these high-end imaging approaches to show the HIV linkage and increase accuracy of their prediction / hypothesis.

*We thank the reviewer for this comment and have performed this experiment in the context of the BlaM assay. We would like to report that we did a BlaM assay with primary cells acutely treated with either 2-DG or a cholesterol-lowering statin (i.e. simvastatin) (please refer to supplemental figure 3). Interestingly, we have been able to show that our results in TZM-bl cells can be recapitulated in primary cells. In particular, acute treatment with 2-DG lead to approximately and 80% reduction in HIV-1_HXB2_ fusion. Furthermore, in primary cells treated with simvastatin, there was a similar reduction in HIV-1_HXB2_ fusion compared to the vehicle control. We have updated the text and have included an additional figure in referenced in lines 222-224 and 300-305 as appropriate.*

For information in line 182, it has been reported that with the endocytosis mediated HIV entry, the cholesterol (at least particle-associated cholesterol) in VSV-G pseudotyped HIV is NOT important for virus entry (reported by multiple labs). On the surface, current interpretation of the authors is not in line with what the field knows. However, this reviewer agrees that whether overall cholesterol levels in the host cells is important for VSV-G pseudotyped HIV can affected endocytosis mediated entry is not known. The main reason (in my opinion) such information is hard to emerge as the pleiotropic effects of such manipulation on the entire cell, making it impossible to properly interpret some of those experiments. As far as I know, there is NO simple / clean way to manipulate cholesterol on PM without affecting other parts of the cells. This assessor feels that the authors are facing similar problems with their claims here on metabolic activity. Unless there is a way to ISOLATE a specific effect on cholesterol regulation at the plasma membrane alone from manipulate metabolic process, proper interpretation would be difficult.

*We thank the reviewer for this comment and appreciate their concern in regards to the data with VSV-G. We agree with the reviewer in that particle-associated cholesterol in VSV-G is dispensable for entry. Our data does not dispute this! This is not the primary aim of our study, and the reviewer has pointed out the knowledge gap in the current field. We aim to determine whether cell-associated cholesterol is important for virus entry, particularly HIV-1. We have illustrated now quite clearly in the text that in primary T cells, T cell lines and in TZM-bl cells that cholesterol lost from the plasma membrane occurs in 2-DG-treated conditions. Furthermore, we show that this loss in cholesterol is also recapitulated in the endosomal compartment. Furthermore, many laboratories have utilized MBCD to remove cholesterol from virus particles and the cell surface to assess the role of cholesterol in virus entry. Although we did not perform this experiment, we disagree with the reviewer and would like to emphasize that there are ways to remove cholesterol from the plasma membrane independently of metabolic activity. The primary reason why we have included VSV-G was to determine if this effect on entry could be seen in other virus envelopes. Clearly, HIV-1_VSV-G_ entry is not as dependent on cholesterol in the host cell as HIV-1_JR-FL_* and this is the point we would like to emphasize in the text, which we have done.

Would have been good if there is a figure number (1, 2, 3, …) in all of the figures and supplement figures. While authors might have label individual figure file with such info, this information is missing in the merged file. This is particular important that all supplement figures are all group together, but there is NO supplement figure 2. For example, there are Figure 1 supplement 1, then jumped to figure 3 supplement 1 and figure 3 supplement 2. This information was not obvious until this assessor has gone through the legends of supplements – unnecessary confusing and annoying! Anything helps reviewers to better understand the paper is a good thing (in my opinion). On the same points, authors are extremely careless in describing and citing their figure number in the text. Very often, author mis-referred to figures, and I was not able to find all figures mentioned. Given this is already a revision, it is a bit concerning.

*We thank the reviewer for this comment and agree that the presentation of the figures can be drastically improved. Compiling all reviewer comments, we have created an updated version of both the main and supplemental figures and hope that these changes appease the reviewers. We have also added figure numbers to all figures in the main and supplemental documents. Furthermore, we have carefully re-examined the text and the figure legends to be explicit when referring to figures and/or supplemental figures. Again, we apologize for the inconvenience.*

Line 186 on (supplement Figure 3B) is not clear. Do author mean Figure 3 supplement 1B or Figure 3 supplement 2B.

*We thank the reviewer for this comment in regards to the labelling of the supplemental figures. Yes, in the older version of the manuscript, the reviewer is correct in stating that supplemental figure 3B is equivalent to Figure 3 Supplement 1B. We have created a more explicit and discrete figure/supplemental figure numbering scheme.*


If authors refer to Figure 3 supplement 1B for comments in Figure 3 supplement 1B, yes, I can see that is statistically significant with *. However, judging from the error bar, it is almost overlapped, and the p-value must be just under 0.05. What if you do that experiment with more biological relevant cells for HIV – ie PBMCs, where entry numbers will be low, and more sensitive to damage by these types of treatments. I wonder what the p value be then.

*We thank the reviewer for this comment and do agree that some experiments would be more appropriate in PBMCs. We have endeavored to perform multiple additional experiments with PBMCs (i.e. BlaM assays in 2-DG-treated conditions, cholesterol quantification, statin-treatment experiments) and hope that the reviewer is satisfied by these experiments and their corresponding results. As the experiment in Figure 3, supplement 1B (now supplemental figure 4) was performed to ensure that a relatively similar phenotypic reduction in HIV-1 fusion could be obtained during glucose deprivation, as opposed to 2-DG treatment, we believe that an addition experiment is not necessary. This is because we have been able to show that even in PBMCs, treatment with 2-DG leads to a reduction in HIV-1 fusion as reported by the BlaM assay.*

*We would also like to stress that the fact that error bars (standard deviation) overlap between different groups of data is not a valid measure to estimate statistically if they are different or not. This is why throughout the text we perform tests based on the analysis of the variance (ANOVA) or the comparison of the mean/s (t Test).* Label in Figure 3 supplement 1C are WAY too small. Again, line 191, wording of ‘supplemental Figure 3C’ was not clear enough for Figure 3 supplement 1C or Figure 3 supplement 2C. Reviewers and audience should not have to guess or to assume.

*We thank the reviewer for rightly pointing out that the text in Figure 3 supplemental 1C was indeed too small. We have endeavored to update all figures to ensure the readability of the text in the figures.*

*We thank the reviewer for this comment in regards to the labelling of the supplemental figure. Yes, the reviewer is correct in stating that supplemental figure 3C is equivalent to Figure 3 Supplement 1C. As stated above, we have created a more explicit and discrete figure/supplemental figure numbering scheme that is hopefully easier to follow for the readers of the manuscript.*

With figure 3 supplement 2c, co-labelling experiment to look at hemifusion using iGFP and DiD dye. Authors should be congratulated on doing these experiments, as they are tough experiments. However, I would suggest the N values of these are limited. Notwithstanding that these are very tough experiments, but from the vantage point of reviewer, I would ask the question whether these numbers are sufficiently significant to demonstrate the claim, rather than the level of difficulty of these experiments themselves.

*We have tracked hundreds of particles to being able to recover 11 independent hemifusion events in the presence of 2DG and 10 full fusion events in the absence of 2DG treatment. These events represent approximately 5- 10% of all events tracked (around 200 for each condition) which is in line with previous reports published by us (Jones and Padilla-Parra 2015 Sci Rep, Padilla-Parra et al., 2013 Cell Rep) or others (Jha et al., 2011 Plos Path; Yamauchi et al., 2009 Cell). Moreover, we have performed these experiments without spinoculation protocols that artificially boost fusion and infection (Jones et al., 2017 Cell Rep). And as demonstrated in Supplementary Figure 4 redirect the point of fusion toward endosomes. The point of the figure is that 2DG treatment arrests fusion at hemifusion. After tracking hundreds of particles we do detect only a few hemifusion events and massively no fusion at all. We have now incorporated the number of total particles tracked in the figure legend to show the relevance of these results.*

Also, authors use TZM-bl system. Why not primary T cells or even T cell line? I seem to recall live imaging of T cells have been done by highly respectable scientists at Oxford some years ago. Would they not be near by the authors’ lab? At least you are all at same University. Furthermore, the upward time (shown) for imaging is 10min that have been done by this team. Understand the nucleus are huge in T cells, BUT since the question is about PM based entry anyway, there will not be a major issue. T-cell experiments would be much more convincing for claim linking with HIV infection.

*As expressed above we have now included fusion data performed with primary cells (Supp Fig. 3). We, in collaboration with the Sattentau lab, indeed in Oxford, did publish some time ago single virus tracking approaches performed in primary fetal macrophages. However, these cells are much flatter than primary T cells and more robust. The challenge when imaging T cells, apart from the big size of the nucleus, is that very fast 3D imaging is needed. When trying these experiments in primary T cells using a confocal system, it causes photodamage and the cells tend to die after a few minutes imaging (as opposed to primary macrophages that turned out to be more resistant as only one confocal plane was sufficient). Other approaches employing multi-colour 3D lattice light sheet microscopy (developed by Betzig) would be more appropriate in that case. Unfortunately, we do not have access to such set-up. Moreover, to our knowledge, and after many searches in pubmed and other sources we could not find any group (apart from ours, Russel et al. 2017) performing real-time single virus tracking in primary cells, for the reasons already discussed. Therefore, in our view, we consider that our data (both functional using single cell imaging BlaM in primary T cells, MT4 and TZM-bl cells and single virus tracking in TZM-bl’s) is enough to support our claims.*

No question that there are many very cool and advance technologies throughout utilised by the authors, but cart should not come in front of the horse. Even with large number of COOL technologies, if biological question cannot be answer properly in the stated claim with the data presented, arguments will fall down. The only exception would be if that is a technological paper to begin with, but this is not the case here. I feel the authors 'overplay' their technology card.

*We thank the reviewer for this comment and appreciate their opinion. We hope that in our ability to address the comments by all reviewers we will be able to resolve this concern so he/she feels comfortable in the claims being made by the paper. This is another reason not to continue with other imaging approaches that would be suitable to address other reviewer’s concerns that ironically would contradict this argument (article too technologically driven).*

*We want to stress, however, that we have employed advanced imaging because these technologies were better suited to test our initial hypotheses and not because we think they are cool. If we were to develop new “cool methods in the context of HIV”, this would be a methods paper, which clearly, is far from being the case.*

From lines 202 to 228, there are A LOT of works here. These are also difficult experiments. I also understand that spin-inoculation will help the number to go up for measurement purpose. Personally, I would have been much more interested to see the ‘Not-Spun’ virus infection experimental data, bright green of Figure 3 supplement 2C. From the text, authors seem to indicate that the data from Main Figure 3C are for non-SPUN virus SVT, perhaps it will be good to remove SPUN virus data from Figure 3 supplement 2 to avoid confusion.

Line 205 ‘Supplemental figure 3D’ meant ‘Figure 3 supplement 1D’ as seen with legend?

*We thank the reviewer for this comment. However, as stated above, we have created a more explicit and discrete figure/supplemental figure numbering scheme that is hopefully easier to follow for the readers of the manuscript.*

Line 215 ‘Supplemental figure 3E’ meant ‘Figure 3 supplement 1E’ as seen with legend?

*We thank the reviewer for this comment. Yes, the reviewer is correct in stating that supplemental figure 3E is equivalent to Figure 3 Supplement 1E. However, as stated above, we have created a more explicit and discrete figure/supplemental figure numbering scheme that is hopefully easier to follow for the readers of the manuscript.*

In ‘Figure 3 supplement 2C’, the distinct categorisation between ‘hemifusion’ and ‘colour separation’ are not clear.

*We thank the reviewer for this comment. Color separation refers literally to the separation of yellow viruses within endosomes (labeled with DiD and Gag-GFP) right at the moment of fusion DiD redistributes around the endosomal membrane and partially uncleaved Gag-GFP is released into the cytosol. (Padilla-Parra et al., 2012 Plos Path).We have now given this definition in the figure legend (Now Supplemental Figure 5).*

According to description on line 213-217, there are two types of DiD dye disappearance events. One is ‘near-asymptotic loss’, while the other one is slow DiD decay. Authors clearly indicated that 7% of rapid decay (colour separation) was previously reported in Vega et al 2011. If I am not mistaken what I have read in the Vega et al paper, the fast decade of DiD (7%) in this case, was referred to be evidence of ‘hemifusion’ event in the last paper (see Vega et al 2011, under Section of ‘Results’, Sub-Section of ‘Blocking HIV-1 uptake by a dynamin inhibitor …’, the last two sentences of Paragraph, and I quote ‘These findings are consistent with the notion that the slow loss of DiD fluorescence is caused by dynasore, whereas the fast DiD decay reflects transfer of viral lipids into the plasma membrane mediated by Env. By definition, lipid mixing in the absence of content release corresponds to a hemifusion phenotype. If these rapid DiD lost event WAS related to hemifusion event before in the Vega paper, why would authors CHANGE the definition NOW that in this MANUSCRIPT that ‘Hemifusion is related to the slow release of DiD dye?

*We did find these two behaviors for single HIV-1 particles undergoing hemi-fusion: 1) fast DiD disappearance and 2) slow DiD disappearance. In both cases these phenotypes do correspond with different hemifusion kinetics. In our opinion, the DiD redistribution within the plasma membrane must be related to hemifusion regardless the kinetics of disappearance. Note that we have absence of photobleaching in our experiments. We ignore why the Melikyan lab has established in de la Vega et al., that slow DiD unmixing is not a proxy for hemifusion. We do not see another explanation for this disappearance of DiD in the absence of artifacts (like quenching or photobleaching, which in our conditions is the case) whilst the GFP-Gag signal remains that these particles undergo hemifusion. For slow DiD disappearance there could be an equilibrium between fusion and fission (Kiss and run) as defined in Zhao et al., 2016 (Nature). In our humble opinion, whatever the case these discussions are out of the scope of the paper.*

Authors MUST clarify the distinction between hemifusion vs colour separation.

*See above.*

Also, given data in Figure 3 supplement 2C that authors clearly have the capacity to distinct different categories of dye disappearance (endosomal fusion, hemifusion, colour separation and plasma membrane fusion), author should (and perhaps MUST) present their main Figure 3C data (with vs without 2DG treatment) that way (ie with these 4 categories - endosomal fusion, hemifusion, colour separation and plasma membrane fusion). These would be MUCH MORE meaningful. It is VERY puzzling that authors have not done that AND make the assumption and I quoted

‘it is extremely likely that the observed hemifusion arrest by 2-DG explicitly occurs in the plasma membrane’ (see line 227-8). This assumption does not seat well with this reviewer. Would authors like to see other manuscript present their major claim of the paper (as seen in abstract and title) basing on an assumption? This is particularly puzzling given the data are within the reach of the authors, and the experiments are high quality.

*We have changed the phrase and now state that according to our labeling strategy hemifusion does occur in the plasma membrane in line 272*

*We have presented the statistics for all events tracked; which indeed were recovered without spinoculation protocols (now specified in the figure legend Supplementary Figure 5). In the presence of 2DG we did not observe either color separation or full fusion (Figure 3C). We present the cumulative distribution of all hemifusion events for this treated cells. Also, we present the data for hemifusion and plasma membrane (PM) fusion for untreated cells.*

On line 223, authors suggested there is (supplement figure 4, related to figure 3). Again, the labelling of figures by authors are poor. I could be wrong, but I was not able to find a supplement figure that is related to spun and non-spun data.

*We thank the reviewer for this comment and hope the new figure labels have solved this issue for the reviewer.*

On line 224, authors claim that PM fusion occurs in a high probability. However, the data in Figure 3 supplement 2 C , there are more endosomal fusion events.

*We now say: Our results illustrate that in non-spun TZM-bl cells, plasma membrane fusion and endosomal fusion may both occur, yet when applying spinoculation protocols, we did not observe plasma membrane fusion events.*

Under section of ‘Single-Cell Metabolic State is linked to … …’, line 230, authors use filipn stain to quantify cholesterol. Stain-ability by filipn is a very indirect way to quantify cholesterol on PM. Why not use mass spec by isolating the membrane fractions?

*We thank the reviewer for this comment and would like to take this space to reassure the reviewer that filipin, in fact, is a very well-established technique to quantify cholesterol on the plasma membrane and in multiple cellular compartments. Kwiatkowska et al. 2014, Wilhem et al. 2017, Kuhnl et al. 2018, and Amini-Bavil-Olyaee et al. 2013 all use filipin to either 1.) quantify cholesterol at the plasma membrane and/or endosomes or 2.) use filipin to quantify cholesterol content in other subcellular structures (e.g. lysosomes). We would like to emphasize here that this list of citations is not exhaustive and there are a plethora of studies published over the last decade that specifically utilize filipin to quantify cholesterol content. Because of these studies and others, we are confident in the data presented.*

*In regards to the reviewer’s suggestion of using mass spectrometry for membrane fraction isolates—although this is an interesting experiment and may perhaps yield data that would only reinforce the data presented in the manuscript in its current form, the goal of the manuscript was to utilize methods that would provide single-cell resolution to the virus-entry process using high-caliber techniques which would inform us the intricacies located at the intersection between cell metabolism and virus entry. Membrane fractionation experiments do not provide near this level of resolution as this approach would only work in bulk (i.e. big population of cells).*

Lipid membrane in PM is dynamics, binding of cholesterol with filipin will OF COURSE affecting PM architecture. This reviewer disagrees with comments made in line 237 by authors. I am surprised authors will cite a book chapter published in 2019 (which I do not have access due to non-open access to verify the authors’ claim from this book). I however do notice that authors have ignored large number of papers and reviews published on lipid rafts and filipin in the past 20+ years. A simple search would have revealed filipin effects on plasma membrane of cells are cell type dependent and concentration dependent. By searching the entire submitted manuscript, authors did not indicate the flipin concentrations used, nor provide control data showing their flipin labelling does not adversely affect the host cells in their hands.

*We thank the reviewer for this comment and would like to refer him/her to the updated methods section of the manuscript. The filipin staining procedure is now located in the methods section in lines 722 to 736. We recognize that we did not clarify how we performed the filipin staining, and therefore would lead to confusion from the reviewer. We would like to stress here that the all of the experiments which required filipin staining were performed after the cells were already fixed and therefore, the filipin staining performed after fixation is unlikely to disrupt the architecture of the cell post-fixation. The reason behind performing filipin staining on fixed cells is because, as the reviewer may have been alluded to, filipin staining is toxic to the cell. This protocol is in accordance with the publications provided above to justify the use of the filipin stain, including the book chapter included from 2019 by Wilhelm and colleagues.*

*We recognize that the comment made in lines 237 therefore may have been confusing to the reviewer and future readers. Therefore, we have revised this accordingly by removing the second half of the sentence as the cells were fixed. We did not realize that the article cited was not accessible by most reviewers and therefore will remove this reference and include references accordingly to establish that filipin is a technique used to quantify cholesterol content in cells.*

*We thank the reviewer also for indicating that the concentration of the filipin stain was not included. We utilized a concentration of 50ug/mL of filipin when performing the filipin staining and this will be updated in the methods section accordingly.*

The MBCD treatments described between lines 239 and 246 are irrelevant to me. In the presence of mixing filipin as a stain, then MBCD, there will be too many effects on the PM, making things way too complicate to interpret. In fact, MBCD will cause cholesterol shuttling between PM and extracellular environment much more rapid.

*We thank the reviewer for this comment and would like to first emphasize that hopefully the above clarifications made for the filipin staining protocol will help guide the reviewer in the context for this experiment. Next, we understand that the reviewer may have some difficulty understanding the use of MBCD. MBCD treatments were performed for multiple reasons: 1.) to provide context as a negative control to ensure the utility of the filipin staining which we used 1mM, 5mM and 10mM MBCD (not shown) and 2.) to provide context as to how much cholesterol was lost via 2-DG treatment. As we do not know the mechanism of cholesterol loss from the plasma membrane in 2-DG-treated cells, our goal with the MBCD treatment was to determine how much MBCD was needed to provide a similar loss in cholesterol from the plasma membrane. We hope that future studies will be able to provide context as to why cholesterol is lost from the plasma membrane.*

*As now stated in the manuscript in the methods section, the filipin staining was performed after any cholesterol depletion (i.e. MBCD) or supplementation (i.e. water-soluble cholesterol) treatments. Therefore, the concern that the reviewer has (i.e. mixing a filipin stain and MBCD) did not occur. The order of the procedure was as follows:*

*TZM-bl cells treated with either a vehicle control, MBCD, or water-soluble cholesterol for 2 hours were fixed with 4% PFA for 15 minutes at room temperature on Ibidi® 35mm gridded dishes. The fixative was washed away three times with cold PBS and then quenched with 1.5mg/mL glycine for 10 minutes at room temperature. Cells were then stained with filipin at 50μg/mL for 2 hours at room temperature, protected from light. The filipin stain was washed away three times with PBS and then cells were subsequently imaged using a SP8–X-SMD microscope with the FALCON module from Leica Microsystems (Manheim, Germany).*

*Because of this, the reviewer should be ensured that the cells were fixed, and any deleterious effect on the plasma membrane after fixation should not have occurred, as all cells were treated with the same concentration of filipin.*

In my opinion, exogenous cholesterol supplement experiment is poorly designed. When exogenous cholesterol is introduced into cell, it will promote cholesterol uptake by cells. If the overall metabolic events of the cell are affected, and impacting on normal cholesterol synthesis then PM targeting, exogenous cholesterol can easily be picked up by host cells from extracellular environment. As a consequence, virus particles such as HIV can readily be picked up – Brugger et al 2006 PNAs already described HIV essentially is a specific type of rafts. Consequently, data in Figure 2C are therefore completely expected for this reviewer – but not for the reason authors thought or claimed.

*Although difficult to interpret, we thank the reviewer for this comment and would like to kindly refer the reviewer to the reagent in question. The cholesterol utilized in this experiment was specifically water-soluble cholesterol, and this is achieved by MBCD-conjugation. Therefore, the cholesterol addition will be supplemented in the plasma membrane only and consequently, the argument made by the reviewer is not accurate. We would like to provide the reviewer with a couple of citations that have used this reagent in the past to supplement cholesterol in the plasma membrane by us: Newton et al 2018, and others: Ringerike et al 2002 (Journal of Cell Science); Carter et al 2009 (Virology).*

Authors indicated there is figure 4D (line 253), but this assessor cannot find figure 4D

*We thank the reviewer for this comment as this has been mentioned by all three reviewers and have now updated the manuscript accordingly.*

This assessor does not know enough about the FliptR dye. However, would one approach be enough to establish an important claim between metabolic events and membrane order? Notwithstanding some of the limitation of the Laurdan, why not use it to provide additional data to strength the claim? In particular, potential coupling with PALM/STORM on CD4 as others have done on LAT (Owen 2012 Nat Comm).

*We thank the reviewer for this comment and recognize that the reviewer is not familiar with the FlipTR dye. The FlipTR dye in this context is vital, owing to the fact that measuring membrane order is more biologically-relevant in living cells. Furthermore, multiple laboratories have utilized this dye since its publication to assess membrane order. Although measuring membrane order via solvatochromism-based methods (i.e. Laurdan). FliptR seems to be more sensitive than Laurdan (a fluorophore originally developed by Gregrio Weber in the late 70’s). In the paper it is clearly stated that FliptR is more sensitive and able to give order information without calibration procedures (Colom et al., 2018 Nat Chem; Riggi et al., 2018 Nat Cell Biol).*

*Although we think that the experiments proposed by the reviewer in regards to PALM/STORM on CD4 would be interesting, we believe these experiments are outside the scope of the paper and are not needed. Furthermore, given that the reviewer has indicated that the technology-aspect of this paper has been utilized heavily, we would not want to further alienate the reader by overloading the paper with multiple advanced microscopy approaches.*

Reviewer #2: (No Response)

Reviewer #3: 1. I believe that Supplemental Figure 1 is still not correct. I think Figures 1C and 1E need to be flipped — they do not correspond to the graphs in 1B and 1C. Also, the figure legends for 1B and 1C do not correspond to the labels in the figure. Especially since the two metabolic reporters show opposite trends, these errors made interpreting Main Figure 1D very hard. However, I think I figured it out and that the data in the main figure make sense.

*We thank the reviewer for this comment and do agree—there are many corrections that were needed to be made with supplemental 1 and we are grateful for his/her keen eye. The reviewer is correct in that the figure legends for 1B and 1C must be flipped to correspond correctly to the figure in question. This has been pointed out by reviewer number 1 and has been corrected accordingly. In addition, the reviewer is correct that there is a problem with supplemental figure 1C and 1E. This has been corrected accordingly, and we apologize for the mistake.*

2. I think the author’s should give better descriptions of their reporter assays. Most HIV readers will understand the assays, but the article should be accessible to others. For example, in Figure 2A, they show the chemical reaction that is catalyzed but don’t explain why a vpr-blam fusion would be a marker for viral entry. The descriptions should be brief but the reader shouldn’t have to go to other references to understand the basis of the assays.

*We thank the reviewer for his/her concern that the reporter assays could be better described. We have now provided descriptions of the B-galactosidase and B-lactamase assay in lines 182-184 and 198-200 respectively in order to aid the reader in interpreting the results stemming from the assay.*

3. With regards to the sometimes poor overlap of cells in the different images in Figure 1 and 2 that the previous reviewer mentioned. The authors arguments make sense, but would it be possible to include transmission images of the cells before and after? It might make it more obvious that the same cells are being imaged and how they have changed. Also, in Figure 2C, it would be helpful if there were images of regions with lower cell density so that the cell identities would be more obvious.

*We thank the reviewer for this comment and appreciate their concern. Unfortunately, transmission images were only taken during the evaluation of the X-gal assay, as this requires a transmission photograph. Therefore, we did not take a before transmission image. The only before images we have taken in this scenario are intensity images and lifetime images. Because of this, not all cells will be visible. We hope the reviewer understands. Nevertheless, the reviewer should feel comfortable that we have endeavored to make sure all cells were identified correctly as the imaging plates we utilized contain grids, which can provide accurate coordinates to a group of cells’ precise location within the dish/well of interest.*

4. Why does the image in Figure 4C w/ 400ug/mL of cholesterol have so many non-infected cells? It appears from the graph that the infection rate should be much higher.

*We thank the reviewer for this comment and this has been corrected accordingly*. *We actually had the images mistakenly labelled, and this has been corrected. We thank the reviewer for these observations.*

5. The authors should consider moving some of the supplemental data into the main figures. Specifically, I found the Flipper-TR data to be clean and convincing and think it should have a more prominent place in the paper.

*We thank the reviewer for this comment and have moved particularly this supplemental figure into the main figure set.*

6. I think that the authors need to address the effects of cholesterol on the endosomal membrane. Even in their “no spin” experiments, a significant portion of fusion events appear to go through the endosome. If they’re trying to correlate infectivity with membrane effects then they need to show convincing data that perturbations that they make to the plasma membrane have similar effects on the endosomal membrane.

*We thank the reviewer for this comment and do agree that an additional analysis of the endosomal compartment would improve the manuscript. We have performed an assay where we have labelled endosomes with LDL-Bodipy and filipin in order to quantify the cholesterol content in the endosomal compartment in both vehicle and 2-DG-treated cells. We have been able to illustrate that there is strikingly a similar reduction in the amount of cholesterol in both the endosomal and plasma membrane compartment. This figure shall be presented as supplemental figure 6C and 6D.*

**Part III – Minor Issues: Editorial and Data Presentation Modifications**
Please use this section for editorial suggestions as well as relatively minor modifications of existing data that would enhance clarity.


Reviewer #1: No additional comments

Reviewer #2: It is worth commenting on whether glycolysis is likely to affect plasma membrane composition beyond cholesterol.

*We thank the reviewer for this comment and agree that glycolysis may in fact lead to a change in plasma membrane composition beyond cholesterol availability. In this regard, we will offer a short commentary in lines 537-543, within the discussion, to suggest additional experiments that should be done to appreciate a more well-rounded understanding on the contribution of glycolytic activity to the availability of plasma membrane lipid composition.*

Why is Figure 1C largely repeated in Figure 2A? This seems unnecessary.

*We thank the reviewer for this comment however, the style of Figure 1C being repeated in Figure 2A is actually vital due to the questions being asked. In particular, Figure 1 and 2 are asking two critically different questions. In particular, Figure 1 is devoted to assessing infection, whereas Figure 2 is devoted to assessing viral fusion. As we utilized a similar imaging-based approach to investigate both phenomena, we resorted to presenting the data in panels similar in Figures 1 and 2 to be cohesive and to guide the readers.*

At several points throughout the figures (e.g. Figure 2B) histograms are presented along side example images. The axes of the histograms are not labeled, and they are not defined in the legend, making them difficult to interpret for the unfamiliar reader.

*We thank the reviewer for this comment and agree that the axes of the histograms should be labelled, and we apologize that they were not labelled previously. We have now amended this concern.*

On line 253, the authors refer to Figure 4D. Figure 4 only has panels A through C.

*We thank the reviewer for this comment and realize this has definitely caused some confusion for all three reviewers. This has been resolved.*

In the model in Figure 6 the hemifusion arrested state is depicted in an unusual way, with the inner leaflet of the viral membrane disrupted. Typically, the inner leaflets are thought to be intact in the hemifusion state. Was this intentional? If not, I would suggest drawing this in the conventional way so as to avoid confusion.

*We thank the reviewer for this comment and can confirm that this has been corrected to show the conventional mechanism of hemifusion.*

Reviewer #3: 1. The G and H figure legends are also swapped in Supplemental Figure 1. In the legend it says that the G graph corresponds to 2-DG, while in the figure it is labeled Oligomycin.

*We thank the reviewer for this comment and this has been corrected accordingly.*
2. Supplemental Figure 2 is mislabeled Figure 3 in the figure legend.

*We thank the reviewer for this comment and this has been corrected accordingly.*

3. I’m assuming in Supplemental Figure 3C that this is not “%” but “fraction”? 0.25% seems very low.

*We thank the reviewer for this comment and have updated the supplemental figures accordingly.*
5. I believe the MT-4 panel of cell images is missing in Figure 4.

*We thank the reviewer for this comment and realize this has definitely caused some confusion for all three reviewers. This has been resolved.*

6. Some of the labels are cutoff in Supplemental Figure 4A.

*We thank the reviewer for this comment and have updated the supplemental figure accordingly.*

7. The authors should label the graphs in Supplemental Figure 5F.

*We thank the reviewer for this comment and have updated the supplemental figure accordingly.*

8. Supplemental Figure 6 is mislabeled Figure 5 in the figure legend.

*We thank the reviewer for this comment and have updated the supplemental figure legend accordingly.*

9. The authors should consider labeling more explicitly the figures themselves. It’s frustrating to have to constantly dig through figure legends to understand what the graphs are showing.

*We thank the reviewer for this comment and have updated the figures accordingly. This issue was similarly pointed out by reviewer number 1 and we agree on its importance. We apologize for the confusion.*
