# Supplementary material for: Correction: Single-cell glycolytic activity regulates membrane tension and HIV-1 fusion
Source: PLoS Pathog. 2021 May 10;17(5):e1009584. doi: 10.1371/journal.ppat.1009584 (PMC8109761; doi:10.1371/journal.ppat.1009584)
Supplement: S2 File — The same method was used to process the data reported in Fig 4C, except that different limits were applied in inverting colors (0–0.9 in Fig 3A, 0–0.8 in Fig 4C), and a different dynamic range was used in obtaining the histograms (0–1.5 in Fig 3A versus 0–3 in Fig 4C). (PDF) [file ppat.1009584.s002.pdf]

Observe that in the article the green-red LUT is inverted to denote fusion + cells in red and fusion - cells in green

To obtain the same images as in the paper:

Open all images in ImageJ
Select Invert LUT
Go to Colors Edit LUT and select the first square to the top-left to black (black background)
Set the limits from 0 - 0.9 for all images in adjust.

To obtain the histograms

Select each image and produce the histograms (Ctrl H) with limits from 0 - 1.5

Observe that this changes do not change the quantitative values for each pixel nor the histogram values

We thought it was more intuitive to present fusion + cells in red
