# Supplementary figures and images for: Correction: Single-cell glycolytic activity regulates membrane tension and HIV-1 fusion
Source: PLoS Pathog. 2021 May 10;17(5):e1009584. doi: 10.1371/journal.ppat.1009584 (PMC8109761; doi:10.1371/journal.ppat.1009584)

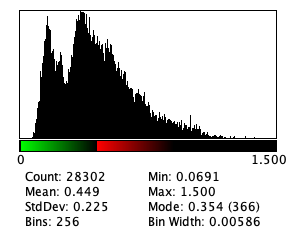

Supplement: S3 File — (ZIP) [file ppat.1009584.s003.zip › LUT images and histograms/Histogram of BlaM_JR-FL_Vehicle.tif]

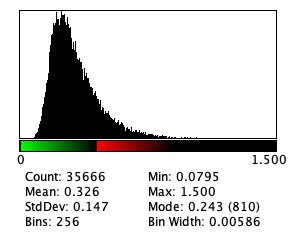

Supplement: S3 File — (ZIP) [file ppat.1009584.s003.zip › LUT images and histograms/Histogram of JRFL_100mM_2DG.tif]

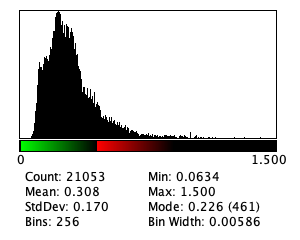

Supplement: S3 File — (ZIP) [file ppat.1009584.s003.zip › LUT images and histograms/Histogram of JRFL_25mM_2DG.tif]

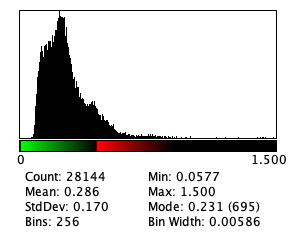

Supplement: S3 File — (ZIP) [file ppat.1009584.s003.zip › LUT images and histograms/Histogram of JRFL_50mM_2DG.tif]

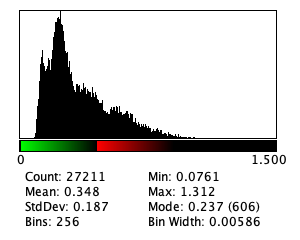

Supplement: S3 File — (ZIP) [file ppat.1009584.s003.zip › LUT images and histograms/Histogram of JRFL_5mM_2DG.tif]

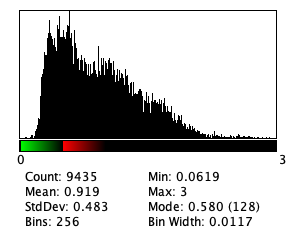

Supplement: S5 File — The corresponding author noted that the histograms in the updated figure differ from those in the published article and were obtained from the same raw image data but applying different analysis thresholds. (ZIP) [file ppat.1009584.s005.zip › TIFF files LUT corrected/Histogram of JRFL_10mMChol_2DG_BlaM.tif]

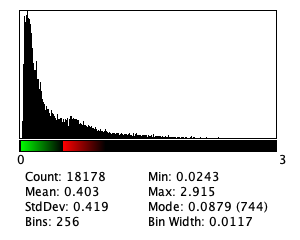

Supplement: S5 File — The corresponding author noted that the histograms in the updated figure differ from those in the published article and were obtained from the same raw image data but applying different analysis thresholds. (ZIP) [file ppat.1009584.s005.zip › TIFF files LUT corrected/Histogram of JRFL_20mMChol_2DG_BlaM.tif]

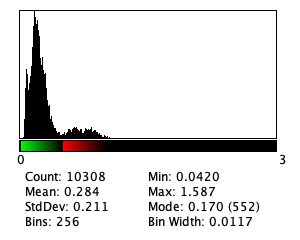

Supplement: S5 File — The corresponding author noted that the histograms in the updated figure differ from those in the published article and were obtained from the same raw image data but applying different analysis thresholds. (ZIP) [file ppat.1009584.s005.zip › TIFF files LUT corrected/Histogram of JRFL_2DG_BlaM.tif]

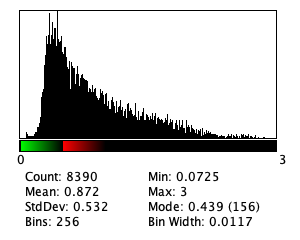

Supplement: S5 File — The corresponding author noted that the histograms in the updated figure differ from those in the published article and were obtained from the same raw image data but applying different analysis thresholds. (ZIP) [file ppat.1009584.s005.zip › TIFF files LUT corrected/Histogram of JRFL_5mMChol_2DG_BlaM.tif]

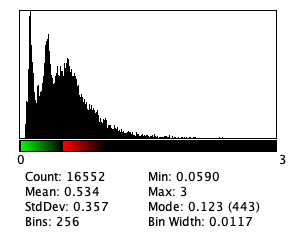

Supplement: S5 File — The corresponding author noted that the histograms in the updated figure differ from those in the published article and were obtained from the same raw image data but applying different analysis thresholds. (ZIP) [file ppat.1009584.s005.zip › TIFF files LUT corrected/Histogram of JRFL_Control_No.tif]

## Slide 1
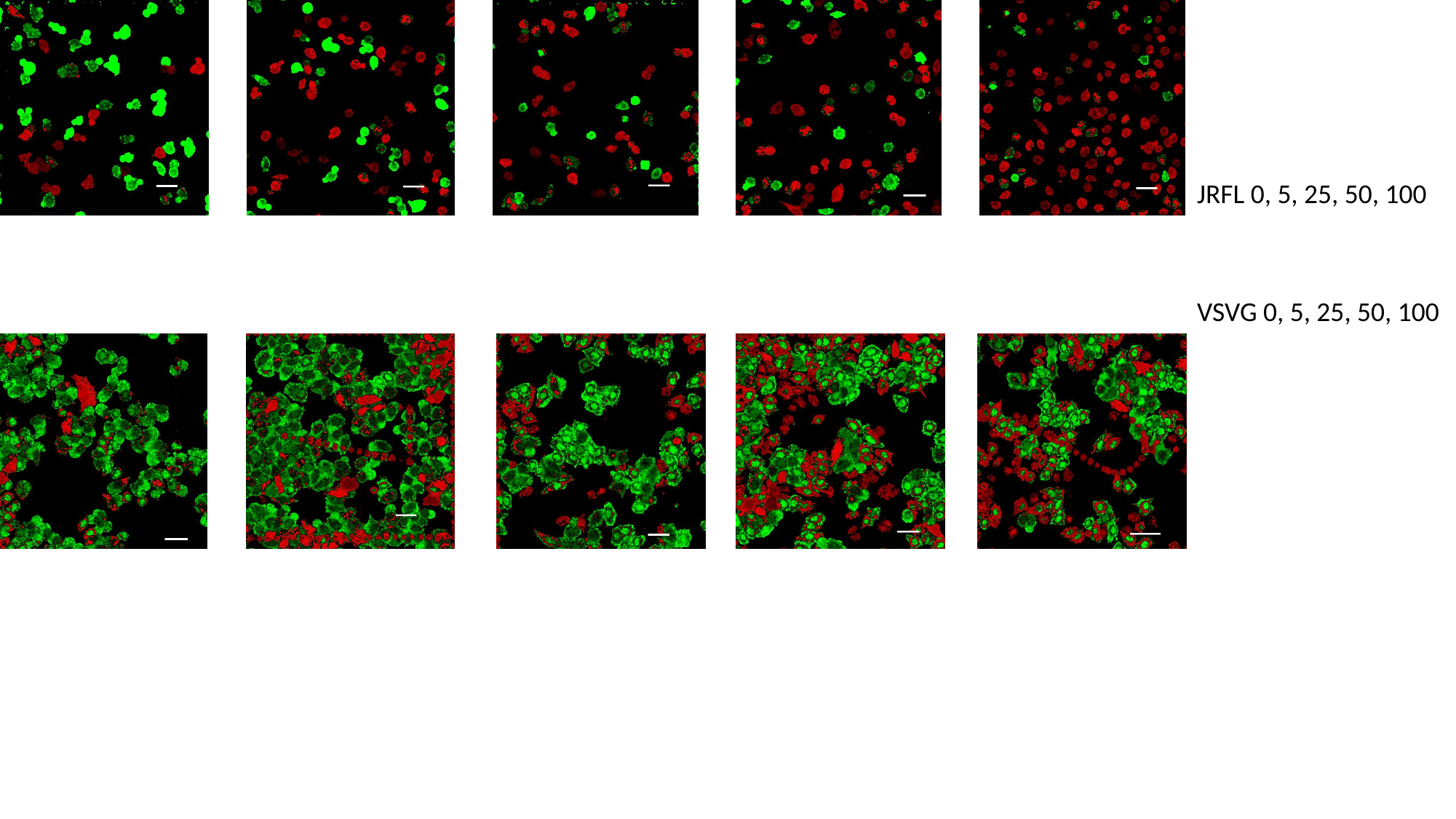

JRFL 0, 5, 25, 50, 100
VSVG 0, 5, 25, 50, 100

Supplement: S6 File — (ZIP) [file ppat.1009584.s006.zip › Figure 3/BlaM Images/Imaging JRFL VSVG 2DG.pptx]
