# Supplementary material for: Correction: Single-cell glycolytic activity regulates membrane tension and HIV-1 fusion
Source: PLoS Pathog. 2021 May 10;17(5):e1009584. doi: 10.1371/journal.ppat.1009584 (PMC8109761; doi:10.1371/journal.ppat.1009584)
Supplement: S6 File — (ZIP) [file ppat.1009584.s006.zip › Figure 3/BlaM Images/Note Fig 3A.pdf]

Observe that in the article the green-red LUT is inverted to denote fusion + cells in red and fusion - cells in green

To obtain the same images as in the paper:

Open all images in ImageJ
Select Invert LUT
Go to Colors Edit LUT and select the first square to the right to black (black background)
Set the limits from 0 - 0..9

To obtain the histograms

Select each image and produce the histograms (Ctrl H) with limits from 0 - 1.5

Observe that this changes do not change the quantitative values for each pixel nor the histogram values

We thought it was more intuitive to present fusion + cells in red
